# Structural Analysis and Conformational Dynamics of *STN1* Gene Mutations Involved in Coat Plus Syndrome

**DOI:** 10.3389/fmolb.2019.00041

**Published:** 2019-06-12

**Authors:** Mohd. Amir, Taj Mohammad, Vijay Kumar, Mohammed F. Alajmi, Md. Tabish Rehman, Afzal Hussain, Perwez Alam, Ravins Dohare, Asimul Islam, Faizan Ahmad, Md. Imtaiyaz Hassan

**Affiliations:** ^1^Centre for Interdisciplinary Research in Basic Sciences, Jamia Millia Islamia, New Delhi, India; ^2^Amity Institute of Neuropsychology and Neurosciences, Amity University Noida, Noida, India; ^3^Department of Pharmacognosy College of Pharmacy, King Saud University, Riyadh, Saudi Arabia

**Keywords:** CST complex protein, STN1, molecular dynamics simulation, OB folds protein, mutational landscape analysis, sequence and structure analysis

## Abstract

The human CST complex (CTC1–STN1–TEN1) is associated with telomere functions including genome stability. We have systemically analyzed the sequence of STN and performed structure analysis to establish its association with the Coat Plus (CP) syndrome. Many deleterious non-synonymous SNPs have been identified and subjected for structure analysis to find their pathogenic association and aggregation propensity. A 100-ns all-atom molecular dynamics simulation of WT, R135T, and D157Y structures revealed significant conformational changes in the case of mutants. Changes in hydrogen bonds, secondary structure, and principal component analysis further support the structural basis of STN1 dysfunction in such mutations. Free energy landscape analysis revealed the presence of multiple energy minima, suggesting that R135T and D157Y mutations destabilize and alter the conformational dynamics of STN1 and thus may be associated with the CP syndrome. Our study provides a valuable direction to understand the molecular basis of CP syndrome and offer a newer therapeutics approach to address CP syndrome.

## Introduction

Telomere is a complex of protein and nucleic acid, crucial for protecting chromosome ends from degradation, end-to-end chromosome fusion, and activation of DNA damage response (DDR) (de Lange, 2009). Abnormal length of a chromosome or deprotected chromosome ends compromise replication potential and thus genome stability (Bodnar et al., 1998). Telomere shortening or degradation are coupled with many human diseases, termed as telomeropathies (Armanios and Blackburn, 2012; Holohan et al., 2014). In addition, the CST complex (CTC1–STN1–TEN1) plays a vital role in the synthesis of C-strand of telomere (Chen and Lingner, 2013) and helps in genome-wide replication and recovery from replication fork stalling during replication stress (Stewart et al., 2012, 2018). The conserved CST complex interacts with the G-strand of the telomere, promotes C-strand synthesis, and suppresses telomerase-mediated elongation of telomere (Chen et al., 2012; Stewart et al., 2012; Feng et al., 2017, 2018). Interruption of CST complex directly affecting the synthesis of C-strand and telomere length leads to the formation of elongated 3′ overhang (Gu et al., 2012).

Regulation of C-strand synthesis and telomere length is primarily regulated by CTC1–STN1 and STN1–TEN1 complex formation (Feng et al., 2018). STN1 consists of an N-terminal OB-fold comprising of a β-barrel, consisting of two- to three-stranded β-sheet sandwiched by three α-helices (Bryan et al., 2013). The N-terminal OB-fold of STN1 forms a strong complex with the OB-fold of TEN1 protein (STN1–TEN1) and thus enables the CST complex for C-strand synthesis. The C-terminal domain of STN1 is composed of β-strands and 11 α-helices organized into two different winged helix-turn-helix (wHTH) motifs (Ganduri and Lue, 2017). Mutations of amino acid residues important for STN1–TEN1 complex formation results in elongated telomeres and several other chromosomal abnormalities including Coat Plus (CP) syndrome (Bryan et al., 2013; Simon et al., 2016).

Owing to the significance of the STN1–TEN1 complex in maintaining telomere homeostasis, mutations in the *STN1* gene are reported to cause CP syndrome. CP is identified by calcification in the intracranial region, hematological abnormalities, and neurologic and retinal defects (Simon et al., 2016). Patients with CP often present shortened telomeres, indicating that telomerase malfunctioning is associated with the pathogenesis. To date, only two STN1 mutations (R135T and D157Y) have been reported that causes CP syndrome. However, the molecular basis of such pathogenesis remained elusive (Simon et al., 2016). In addition, *in vitro* mutational analysis in the *STN1* and *TEN1* gene depicts that mutation in the *TEN1* gene, in particular R27Q, Y115A, and R119Q, shows a marked change in their dissociation constant. However, STN1 double mutants (D78A/I164A and D78A/M167A) show a complete loss of binding with TEN1 (Simon et al., 2016).

Herein, we have analyzed the complete mutational spectrum in the *STN1* gene to identify the disease-causing mutations and subsequent pathogenic characterization based on their impact on structure and functions. To understand the molecular basis of CP syndrome, the structural and conformational changes in R135T and D157Y mutants were extensively studied at an atomic level using 100 ns molecular dynamics (MD) simulation. The results possibly unveil an understanding of R135T and D157Y mutations and their association with the CP syndrome.

## Materials and Methods

### Collection of Dataset

FASTA sequence of STN1 was retrieved from the UniProt database (UniProt ID: Q9H668). Distribution of SNPs was collected from Ensembl (Hubbard et al., 2002), dbSNP (Sherry et al., 2001), and OMIM (Amberger et al., 2008) databases. Functional annotation of each SNP was extracted from the dbSNP database. Structures of STN1 were obtained from the Protein Data Bank (PDB code: 4JOI and 4JQF) (Berman et al., 2000).

### Prediction of Deleterious nsSNPs

Deleterious or damaging nsSNPs in the *STN1* gene were predicted by utilizing Sorting Intolerant from Tolerant (SIFT) (Kumar et al., 2009), PolyPhen 2.0 (Adzhubei et al., 2013), and PROVEAN (Choi and Chan, 2015) web servers. SIFT predictions are based on the sequence homology and it differentiates nsSNPs as tolerant (neutral) or intolerant (disease) on the basis of a predicted score (deleterious if a score is <0.05 and neutral if a score >0.05). PolyPhen 2.0 calculates the impact of point mutations on the structure of protein as well as its effects on phenotype. A detailed description of methods for deleterious nsSNP prediction is provided in our earlier communication (Amir et al., 2019).

### Prediction of Destabilizing nsSNPs

Protein stability is represented by the change in the Gibbs free energy (Δ*G*) upon folding. In this study, we have utilized five stability prediction tools, DUET (Pires et al., 2014), SDM2 (Pandurangan et al., 2017), mCSM (Pires et al., 2013), CUPSAT (Parthiban et al., 2006), and STRUM (Quan et al., 2016). DUET is an integrated computational method used for calculating the impact of mutations on the stability of the protein. SDM2 differentiates the competence of amino acid residues between the WT and mutant proteins, whereas the mCSM algorithm establishes effects of mutations depending on the structural signature. CUPSAT uses protein structure environment-specific potential and torsion angle potential to find the differences in stability of WT and mutant proteins. It requires a PDB structure and the position of the substituted residue. The output entails information about its structural features, sites of mutation, and inclusive information about the change in the stability of protein. STRUM calculates the impact of mutations using conservation score obtained from alignment of the multiple-threading template.

### Prediction of Pathogenic nsSNPs

MutPred2 is a computational server used to predict the molecular basis of disease-associated substitutions. It employs many attributes such as protein structure, its function, and evolution. MutPred2 utilizes three different servers, PSI-BLAST (Altschul et al., 1997), SIFT (Ng and Henikoff, 2003), and Pfam (Finn et al., 2015) together with some structural disorder prediction methods such as MARCOIL (Delorenzi and Speed, 2002), TMHMM (Krogh et al., 2001), and DisProt (Sickmeier et al., 2006). Thus, MutPred combines the score of these computational servers and then provides prediction results. PhD-SNP is a single-sequence SVM-based tool that distinguishes disease-associated mutations depending on the local environment of substitution (Capriotti et al., 2006).

### Aggregation Propensity Analysis

Solubility is an important feature required for the function of a protein. Predicting solubility of a particular protein is critical for protein engineering and normal functioning. SODA is a newly developed aggregation prediction method (Paladin et al., 2017). It predicts the change in protein solubility based on a number of physicochemical features. SODA utilizes the propensity of amino acid sequence to aggregate, intrinsic disorder, secondary structure, and hydrophobicity to calculate the differences in protein solubility (Paladin et al., 2017).

### Structure Refinement

The atomic coordinates of the STN1 structure were retrieved from the STN1–TEN1 complex available in the Protein Data Bank (PDB ID: 4JOI). The STN1–TEN1 complex comprises of the N-terminal domain of STN1 (residues 24–184) and full-length TEN1 (residue 2–124). The structure of the N-terminal domain of STN1 has a missing loop (residues 92–110) that was modeled using MODELER 9.20 imbedded in PyMOL plugin PyMod 2.0. Further, the WT N-terminal domain of STN1 was used to create R135T and D157Y mutations using mutagenesis plugin embedded in PyMOL (www.pymol.org). Mutant structures and the WT structure of STN1 were energy-minimized using SPDBV to remove high energy configurations by changing their coordinate geometries in such a way as to release internal constraints and reduce the total potential energy.

## Molecular dynamics simulations

To in-depth understand the effects of mutations on the STN1 structure, all-atom MD simulations were carried out for 100 ns under explicit water solvent conditions for WT and its mutants (R135T and D157Y). MD simulations were performed at 300 K at the molecular mechanics level implemented in the GROMACS 5.1.2 software package using the GROMOS96 43a1 force field. All systems were soaked in a cubic box of water molecules with a dimension of 10 Å using the *gmx editconf* module for setting boundary conditions and *gmx solvate* module for solvation. Further, the systems were subsequently immersed in a box having a simple point charge (SPC16) water model. Na^+^ and Cl^−^ ions were aided further in the systems for neutralizing and preserving a physiological concentration (0.15 M) using the *gmx genion* module. All the systems were minimized using 1,500 steps of steepest descent. All systems were equilibrated at a constant temperature, 300 K, by utilizing the two-step ensemble process (NVT and NPT) for 100 ps. Initially, the Berendsen thermostat with no pressure coupling was employed for the NVT (i.e., constant number of particles, volume, and temperature) canonical ensemble, and then we use the Parrinello–Rahman method pressure of 1 bar (P) for the NPT ensemble (i.e., constant particle number, pressure, and temperature). The final simulations were performed for each system for 100 ns where leap-frog integrator was applied for the time evolution of trajectories. The details of MD simulations have been described elsewhere (Gulzar et al., 2018; Naqvi et al., 2018).

### Analysis of MD Trajectories

All the trajectory files were analyzed using trajectory analysis module embedded in the GROMACS simulation package and Visual Molecular Dynamics (VMD) software. The trajectory files were analyzed by using *gmx confirms, gmx rmsd, gmx rmsf* , *gmx gyrate, gmx sasa, gmx hbond, gmx covar, gmx anaeig, gmx energy, gmx do_dssp*, and *gmx sham* GROMACS utilities to extract the graph of root-mean-square deviation (RMSD), root-mean-square fluctuations (RMSFs), radius of gyration (*R*_g_), solvent accessible surface area (SASA), hydrogen bond, principal component, secondary structure, etc. (Syed et al., 2018). All the graphs were plotted using the Qt Grace Visualization tool.

### Principal Component and Free Energy Landscape Analysis

Principal component analysis (PCA) was done to study the collective motions of WT and mutant STN1. This method employs the calculation and diagonalization of the covariance matrix. The covariance matrix is calculated as:

$$\begin{array}{r} C_{ij}=<\left(x_{i}-<x_{i}>\right)\left(x_{j}-<x_{j}>\right)> \end{array}$$

where x_*i*_/x_*j*_ is the coordinate of the *i*th/*j*th atom of the systems, and < —> represents an ensemble average.

Free energy landscape (FEL) can be used to understand the stability, folding and function of the protein. The FEL can be constructed as:

$$\begin{array}{r} \Delta G\left(X\right)=-K_{\text{B}}T\;ln\;P\left(X\right) \end{array}$$

where *K*_B_ and *T* are the Boltzmann constant and absolute temperature, respectively, and *P*(*X*) is the probability distribution of the molecular system along the PCs.

## Results and Discussion

This study provides the structural and functional consequences of nsSNPs in the *STN1* gene. In addition, disease-causing or pathogenic spectrum, aggregation behavior, and conservation score were screened using advanced computational methods. Finally, the atomistic levels of two pathogenic mutations (R135T and D157Y) causing CP syndrome have been analyzed in detail using all-atom MD simulation approach.

### Prediction of Deleterious and Destabilizing nsSNPs in *STN1* Gene

**Figure 1:**
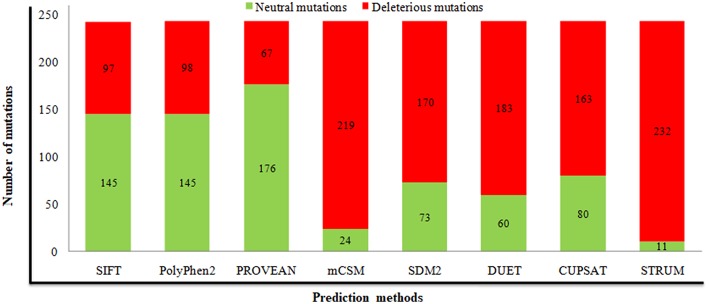
Prediction of deleterious and destabilizing mutations using different computational methods. Numbers of neutral and deleterious mutations are indicated in green and red, respectively.

For prediction of deleterious and destabilizing nsSNPs, we have cross-checked information present in the dbSNP and UniProt databases, removed invalid mutations based on wrong amino acid position and alignment, and merged or removed data with other nsSNPs in dbSNP. As a result, a sum of 243 nsSNPs in the *STN1* gene was considered for analysis (Tables S1, S2). Distribution of deleterious nsSNPs in *STN1* gene using SIFT, PolyPhen 2.0, and PROVEAN predicated that 97 (~40%), 98 (40%), and 67 (27%) nsSNPs were found deleterious, respectively (Figure 1 and Table S1). Similarly, predictions of destabilizing nsSNPs employing CUPSAT 163 (67%), SDM 170 (70%), DUET 183 (75%), mCSM 219 (90%), and STRUM 232 (95%) nsSNPs depict a destabilizing effect (Figure 1 and Table S2). The nsSNPs that depict a deleterious effect by at least two different tools and the destabilizing behavior by at least three different methods were collected for phenotypic spectrum analysis. A total of 76 nsSNPs were taken for this analysis.

### Analysis of Molecular Phenotype

Molecular phenotype analysis complements the characterization of disease-associated nsSNPs in the human genome. All 76 deleterious and destabilizing nsSNPs were subjected to MutPred 2.0 and PhD-SNP computational servers to find the relation of these deleterious and destabilizing nsSNPs with the disease phenotype. Prediction of disease phenotype using MutPred 2.0 and PhD-SNP shows that 39 (51%) and 41 (53%) nsSNPs are linked with disease phenotype, respectively (Table S3). Further, about 30 (39%) mutations identified as disease-causing from both prediction methods were further used for aggregation propensity scanning.

### Aggregation Propensity Analysis

The solubility of protein is one of the important characteristics that primarily belong to the concentration, conformation, and location of the protein. It plays a critical role in protein turnover in the cells (Ross and Poirier, 2004; Sami et al., 2017). Protein aggregation often tends to the progression of a range of pathologies such as Alzheimer's (Thal et al., 2015) and Parkinson's (Tan et al., 2009). Analysis of aggregation behavior using the SODA web server aids in molecular identification of a disease or pathogenic nsSNPs to the conformational level. Of 30 pathogenic mutations identified using MutPred and PhD-SNP prediction servers, 10 mutations (G45D, G51V, C88Y, R135T, D157Y, P158S, R166G, Y174C, G278R, and C312F) show a decrease in the solubility score (Table 1). Results depict that majority of aggregate-forming mutations lie in the N-terminal domain of STN1 in comparison to the C-terminal domain.

**Table 1:** Predictions of aggregation propensities of pathogenic mutations in the *STN1* gene.

**S. No**.	**Variants**	**Aggregation**	**Disorder**	**SODA**	
				**Score**	**Solubility**
1.	Wild-type	−4.811	0.061		
2.	L24P	21.835	−0.045	18.9	More soluble
3.	F26L	2.418	0.151	2.804	More soluble
4.	K28E	9.961	0.119	10.098	More soluble
5.	L29H	8.971	−0.034	10.035	More soluble
6.	L35R	7.737	−0.342	8.774	More soluble
7.	G45D	−1.32	−0.142	−1.864	Less soluble
8.	G51V	−45.514	−0.156	−45.385	Less soluble
9.	I54M	9.016	−0.105	9.137	More soluble
10.	F72L	2.259	0.197	2.922	More soluble
11.	Y73N	6.435	0.693	7.166	More soluble
12.	I84K	42.824	−2.025	38.395	More soluble
13.	C88Y	−10.812	−0.99	−11.988	Less soluble
14.	**R135T**	−36.68	0.636	−35.951	Less soluble
15.	**D157Y**	−17.547	0.943	−15.669	Less soluble
16.	P158S	−12.077	0.057	−11.033	Less soluble
17.	R166G	2.119	−0.408	−1.136	Less soluble
18.	I173N	13.237	−0.614	10.934	More soluble
19.	Y174C	2.797	−2.037	−1.837	Less soluble
20.	L216H	0.393	−0.096	0.141	More soluble
21.	F224S	7.258	−0.824	3.977	More soluble
22.	G278R	−4.977	−0.001	−2.807	Less soluble
23.	Y291C	2.76	−0.023	2.24	More soluble
24.	L300P	5.612	0.16	0.421	More soluble
25.	I304N	19.714	0.214	15.114	More soluble
26.	C312R	1.177	0.99	4.51	More soluble
27.	C312F	−0.777	0.01	−0.593	Less soluble
28.	C322Y	2.059	−0.046	2.91	More soluble
29.	H326P	27.065	−0.019	22.52	More soluble
30.	Y365N	12.673	0.709	12.761	More soluble
31.	Y365C	2.461	−0.131	1.773	More soluble

### Sequence Conservation Analysis

**Figure 2:**
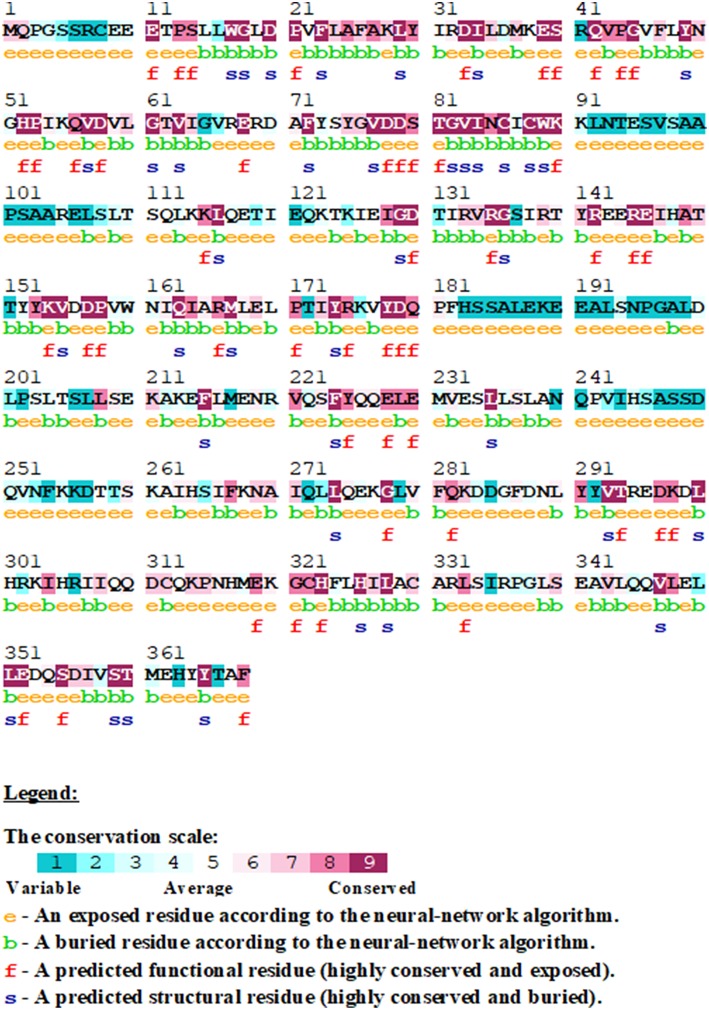
Sequence conservation analysis in STN1 protein using ConSurf. The conservation score from highly variable to conserve is represented in a scale of 1–9.

Analysis of conserved amino acid residues in protein provides a better understanding of the significance of a particular amino acid residue and probably its localized evolution. Sequence conservation analyses in the STN1 protein have been performed using the ConSurf tool, which depicts that most of the amino acid residues in the STN1 protein are highly conserved (Figure 2). In particular, the stretches of highly conserved amino acid residues such as 23–30, 42–45, 56–58, 60–65, 75–86, 88–90, 153–155, 173–175, 178–180, 228–230, and 321–323 were found. From this conservation analysis, it is very clear that the N-terminal domain of STN1 is considerably more conserved than the C-terminal domain. The stretches of variable residues in the STN1 protein had the following ranges: 2–10, 90–120, 180–207, and 230–260. Variable stretches are usually long and mainly concentrated in the middle of protein length compared to conserved stretches, which are usually short and scattered throughout the length of the STN1 protein.

### Association of STN1 Domains With Disease-Causing Mutations

**Figure 3:**
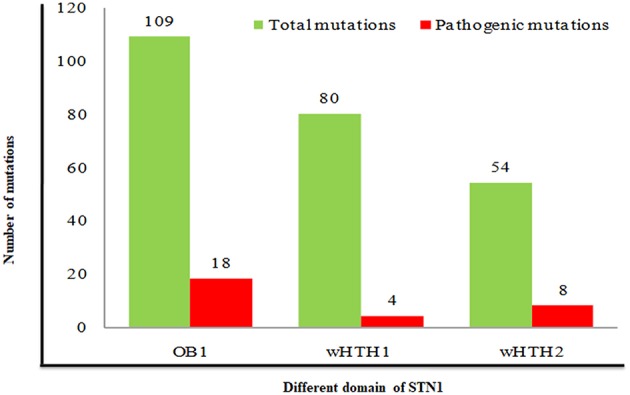
Association of pathogenic mutations in different domains of STN1 protein.

Analysis of pathogenic/disease-causing mutations in different domains of the *STN1* gene entails relative information about a particular domain to be disease-causing or neutral. Distributions of pathogenic mutations were analyzed in all three domains (OB1, wHTH1, and wHTH2) of STN1. We have calculated the percentage of pathogenic mutations in each domain by dividing total mutation occurring in a particular domain to the pathogenic mutations found in the same domain. The nsSNPs in OB1, wHTH1, and wHTH2 domains have a 16%, 5%, and 14% chance of being pathogenic, respectively (Figure 3). These results depict that the N- and C-terminal domain of STN1 (OB1 and wHTH2) are possibly more prone to pathogenesis. These interpretations are further complemented by a high conservation score of the N- and C-terminal domain of STN1 in contrast to the wHTH1 domain. How does a particular mutation disrupt the structure of STN1 and its function? To answer this, we have performed 100-ns MD simulation of R135T and D157Y, to understand the molecular mechanism of the disease.

### Mutations Induced Conformational Stability, Flexibility, and Dynamic Changes

**Figure 4:**
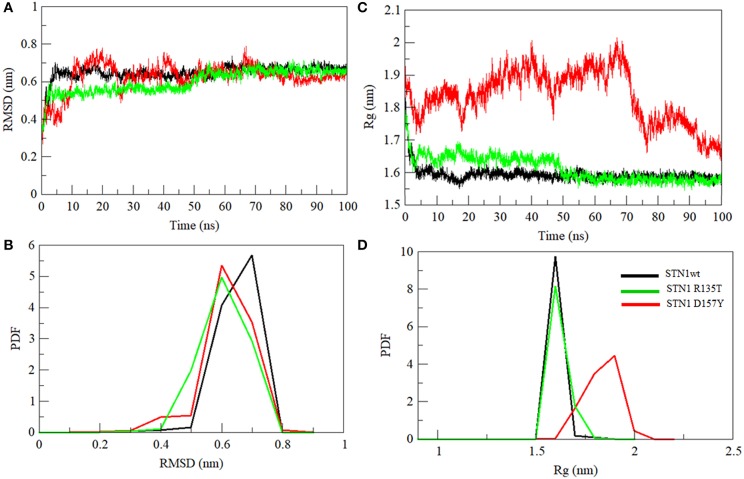
Conformational changes in STN1 protein and its mutant. **(A)** Graphical representation of backbone conformation. **(B)** Probability distribution functions of RMSD. **(C)** Radius of gyration. **(D)** Probability distribution functions of the compactness of wild-type and STN1 mutants. Wild-type (black), R135T (green), and D157Y (red).

MD simulation was employed to investigate the disruptive effects of R135T and D157Y (run in duplicate) mutations on the conformational stability of STN1 through 100-ns MD simulations. The initial and final structures of WT, R135T, and D157Y during simulation runs are shown in Figure S1. We did not find any significant structural changes in WT except for the changes in the loop connecting strand β3 and β4, which become more flexible after 100 ns of simulation. Interestingly, loss of the C-terminal helix structure was observed in the case of D157Y. The final structures obtained at 100-ns simulation were superimposed and are shown in Figure S2. The figure clearly shows that there is significant change upon D157Y mutation as the RMSD between WT and D157Y structures is 3.51 as compared to R135T with an RMSD of 1.96. Overall changes in the STN1 stability upon mutations were investigated by RMSD calculation. It depicts the differences in backbone flexibility of C^α^ atoms of WT and mutants. The average RMSD values for WT, R135T, and D157Y mutations were calculated as 0.64, 0.62, and 0.60 nm, respectively (Table 2). These results indicate no significant differences in the RMSD values of mutants (Figure 4A). Moreover, we did not find any significant changes in any of the structure parameters of two simulation runs in D157Y (Figure S3). For consistency, the probability distribution function (PDF) of WT conformers was confined within a range of 0.5–0.8 nm, whereas mutations R135T and D157Y conformers lie within a range of 0.4–0.8 and 0.3–0.8 nm, respectively (Figure 4B). These results also indicated no remarkable differences in the values of PDF of the WT and mutants.

**Table 2:** The calculated parameters for all the systems obtained after 100-ns MD simulations.

**S. No**.	**Protein**	**Average RMSD (nm)**	**RMSF (nm)**	**Radius of gyration (nm)**	**Average SASA (nm^**2**^)**	**Average no. of HB**
1.	WT	0.64	0.19	1.59	92.42	114
2.	STN1 D157Y	0.62	0.31	1.83	99.02	105
3.	STN1 R135T	0.60	0.21	1.61	93.43	106

Compactness of WT and STN1 mutants is denoted by *R*_g_, which represents the compactness of the protein structure and is associated with stability (Lobanov et al., 2008). Differences in *R*_g_ values between WT and mutants are shown in Figure 4C. Average *R*_g_ values of WT, R135T, and D157Y mutations were calculated as 1.59, 1.61, and 1.83 nm, respectively (Table 2). The mutation D157Y shows a significant increase in *R*_g_, suggesting a loss in compactness. PDF analysis further suggested a higher probability of *R*_g_ value of D157Y compared to R135T and WT (Figure 4D).

**Figure 5:**
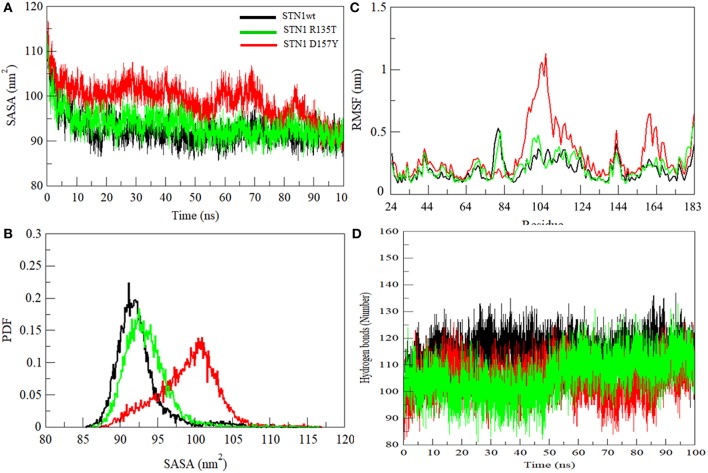
**(A)** Graphical representation of SASA. **(B)** Probability distribution of SASA. **(C)** Residual fluctuations. **(D)** Intra-protein hydrogen bond analyses of wild-type (black), R135T (green), and D157Y (red) mutants of STN1.

We have also investigated the hydrophobic core region of WT and both mutants by calculating the change in solvent accessibility surface area (SASA). A significant increment in average SASA has been observed in the D157Y mutant (Figure 5A and Table 2). However, R135T mutation does not show any significant change in the SASA value compared to WT as Arg135 is located on the surface of the protein. D157Y mutation brings larger changes in the SASA value because Asp157 is partially buried and replacement of Asp157 by Tyr renders this residue more accessible to solvent. The increase in average SASA value and PDF suggested that D157Y has a large surface exposed to solvent, and this might cause the exposure of hydrophobic residues and subsequently unfolding of the protein (Figure 5B).

Residual flexibility of WT and mutants was estimated and is shown in Figure 5C. It is evident that the D157Y mutation reduces the fluctuations of Asp78, which is implicated in salt bridge formation with the residue of TEN1 (Bryan et al., 2013). Disruption in salt bridge formation between Asp78 of STN1 and Arg27 of TEN1 results in loss of about 5.5-fold affinity between both proteins. A significant increase in fluctuations of residues from 94 to 124 was observed. These residues, which are highly non-conserved and belong to the α2 helix, lie between strands β5 and β6 of STN1 (Figure 2). The exact function of the α2 helix is not known possibly due to partial visibility of the electron density map (Bryan et al., 2013). Moreover, we observed the increase in the fluctuations of residues 156–174 belonging to the α3 helix of STN1, which is implicated in hydrophobic interaction with residues of the α2 helix of the TEN1 protein. An interruption in the formation of a hydrophobic interaction of these residues reduces the interaction of STN1 and TEN1 with binding affinity ranges from 2.5- to 5.5-fold (Bryan et al., 2013). Thus, Asp78 forms a salt bridge with Arg27 of TEN1. This salt bridge plays a critical role for proper functioning of CST complex. We have analyzed the change in deviation, compactness, and SASA of Asp78 during the course of simulation (Figure S4).

Hydrogen bond (HB) analysis in WT and STN1 mutants is essential to understand the stability and flexibility of the protein. Figure 5D illustrates the number of intramolecular HB network in the WT and mutant STN1 protein during the course of simulation. The number of HB decreases in R135T and D157Y mutants as compared to the WT (Table 2). WT STN1 has an average of 114 HB, whereas R135T and D157Y mutants have an average of 105 and 106 HBs, respectively. This reduction in HB reflects a loss of structure and compactness of the STN1 protein. Overall, D157Y mutation disrupts the structure, stability, and dynamics of STN1, which leads to interruption of STN1 and TEN1 interaction and thus results in telomere signal free ends and elongated and fragile telomeres, acquiring phenotypes related to telomere length deregulation and dysfunctional CST complex.

### Secondary Structure Analysis

**Figure 6:**
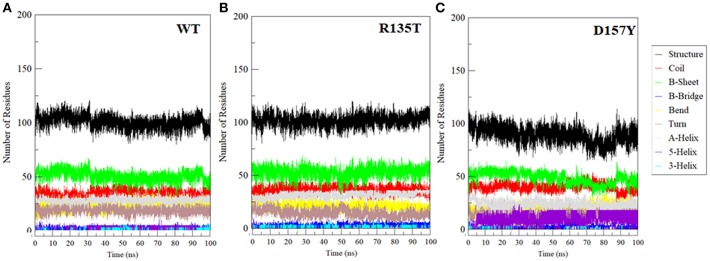
Secondary structure changes during the course of 100-ns MD simulation in **(A)** wild-type, **(B)** R135T, and **(C)** D157Y mutants.

The propensity of secondary structural content is a crucial component to study the structural behavior of the protein. We have investigated the changes in secondary structure in WT and R135T and D157Y mutants as shown in Figure 6. The mutation R135T does not induce any significant change in the secondary structure content (Figure 6B), while a large decrease in overall structure was noticed in the case of the D157Y mutant (Figure 6C). The large changes in secondary structure content in the D157Y mutant are consistent with the results obtained from other conformational analysis. Our MD analysis has clearly indicated that the D157Y mutation disrupts the conformational stability, dynamics, and flexibility of STN1, which may be a possible cause of CP syndrome.

### Principal Component and Free Energy Landscape Analysis

**Figure 7:**
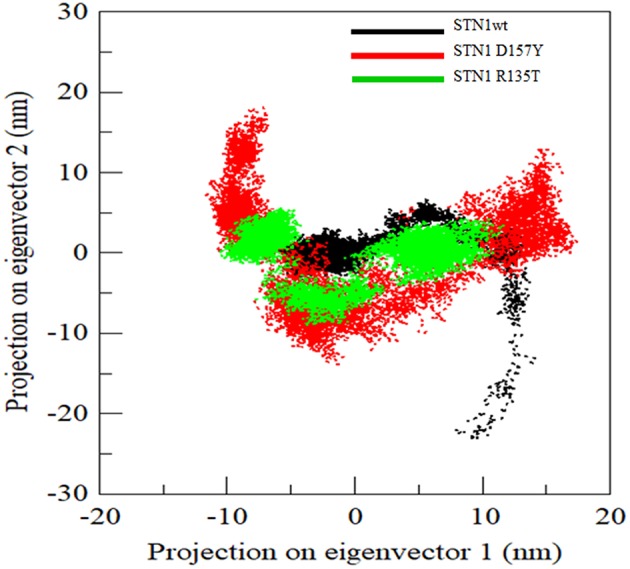
Projection of C^α^ atoms in essential subspace along the first two eigenvectors of wild-type (black), R135T (green), and D157Y (red) mutant of STN1.

Generally, proteins carry out their specific functions through collective atomic motions. Hence, a collective atomic motion of a particular protein is used as a parameter to understand the stability of protein. PCA is used to investigate the global motions of protein into a few principal motions, characterized by eigenvectors and eigenvalues. Figure 7 shows the conformational sampling of WT and mutant SNT1 in the essential subspace by projecting the C^α^ atom, showing the tertiary conformations along eigenvectors 1 and 2. From the projection of PC1 and PC2 of WT and mutant, we could envisage a cluster of stable states in WT and mutants. Results clearly depict that the mutants cover a wide range of phase spaces as compared to WT, especially D157Y. The large increase in overall motion in D157Y might be considered as the prime cause in the impairment of protein function.

**Figure 8:**
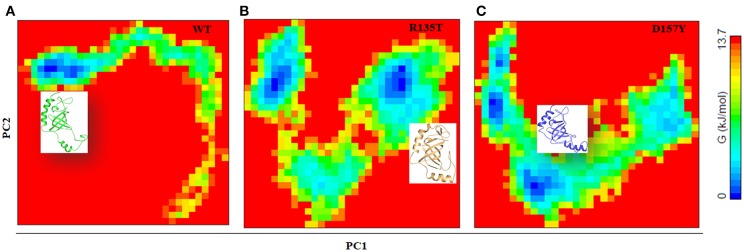
Free energy landscape analysis of **(A)** wild-type, **(B)** R135T, and **(C)** D157Y mutants of STN1 along with the structural snapshots present in the stable minima of each FEL.

To distinguish the conformational states of WT and mutant STN1, the Gibbs free energy landscape (FEL) was calculated using the first two principal components as reaction coordinates. Using PCA, Helmoltz free energy change is calculated and the FELs thus obtained from the simulations are plotted, as shown in Figure 8. The FEL can provide remarkable information about the different conformational states accessible to the protein in the simulation. Figure 8 shows the FEL of (A) WT and its mutants (B) R135T and (C) D157Y along the two principal components. As can be seen from the figure, WT STN1 displayed a well-defined single large global energy minima basin associated with its conformational state. R135T, in addition to native energy minima, explores wide global energy minima through a transition state. This energy minimum corresponds to a structure very much similar to WT STN1 with some loss of irregular secondary structure like bend and turn.

Interestingly, in D157Y, native basin splits into two relative energy minima, suggesting the destabilization of native state. The presence of multiple energy minima in the conformational space achieved in D157Y indicates the significant destabilization of the protein. Moreover, the structural snapshots revealed the presence of a structure with significant loss of C-terminal helices α3 along with minor loss of β2 and β3 residues with the overall increase in coil structure.

## Conclusions

Here, we have investigated the effect of deleterious or destabilizing mutations in the STN1 protein that are presumably associated with the CP syndrome. Mutational landscape analysis suggested that about one-third of nsSNPs in the *STN1* gene are deleterious, and a high rate of pathogenic occupancy was found in the OB1 domain in contrast to the wHTH1 and wHTH2 domains. Backbone conformation, residual flexibility, compactness, solvent accessibility, hydrogen bond, secondary structure, protein motion, and FEL analyses clearly indicated the significant structural and functional loss in the STN1 protein due to D157Y mutation. However, mutation R135T behaves similar to the WT. In conclusion, our results provide an in-depth understanding of destabilization and loss of conformational dynamics of STN1 mediated by D157Y mutation. The results may thus be further exploited to understand the cause of CP syndrome and development of novel strategies for the therapeutic management of CP.

## Author Contributions

MA and MH: conceptualization and project administration. MA, TM, VK, and RD: methodology. MA, MR, AH, MFA, PA, and RD: software. TM, AH, MFA, AI, and MH: validation. TM, VK, MR, and FA: formal analysis. MA, TM, VK, and MH: investigation and writing—original draft preparation. MFA, AH, and MH: resources. MA, MR, and AH: data curation. MFA, AI, and FA: writing—review and editing. TM: visualization. MH: supervision. MFA and MH: funding acquisition.

### Conflict of Interest Statement

The authors declare that the research was conducted in the absence of any commercial or financial relationships that could be construed as a potential conflict of interest.
