# Supplementary material for: Structural Analysis and Conformational Dynamics of STN1 Gene Mutations Involved in Coat Plus Syndrome
Source: Front Mol Biosci. 2019 Jun 12;6:41. doi: 10.3389/fmolb.2019.00041 (PMC6581698; doi:10.3389/fmolb.2019.00041)
Supplement: Supplementary file 1 [file Table_1.DOCX]

**Table S1:** Sequence-based identification of deleterious/damaging mutations in *STN1* gene.

| **S. No.** | **Variant ID** | **Variants** | **SIFT** | | **PolyPhen-2** | | **PROVEAN** | |
| --- | --- | --- | --- | --- | --- | --- | --- | --- |
|  |  |  | **Score** | **Remark** | **Score** | **Remark** | **Score** | **Remark** |
|  | rs757883251 | L24P | 0.19 | tolerated | 0.548 | possibly damaging | -2.523 | deleterious |
|  | rs1276684506 | A25G | 0 | deleterious | 0.955 | probably damaging | -2.506 | deleterious |
|  | rs1231230029 | F26L | 0 | deleterious | 0.918 | probably damaging | -3.467 | deleterious |
|  | rs1318935234 | K28E | 0 | deleterious | 0.152 | benign | -2.756 | deleterious |
|  | rs769177803 | L29H | 0 | deleterious | 0.999 | probably damaging | -5.589 | deleterious |
|  | rs749313928 | L35R | 0.01 | deleterious | 0.687 | possibly damaging | -3.733 | deleterious |
|  | rs780153128 | D36N | 0.14 | tolerated | 0 | benign | -0.470 | neutral |
|  | rs769872828 | M37V | 0.01 | deleterious | 0.001 | benign | -1.608 | neutral |
|  | rs1271998118 | K38T | 0.22 | tolerated | 0.131 | benign | -1.089 | neutral |
|  | rs931263178 | R41S | 0.65 | tolerated | 0.048 | benign | -0.616 | neutral |
|  | rs373416912 | Q42H | 0 | deleterious | 0.986 | probably damaging | -2.644 | deleterious |
|  | rs780839582 | V43L | 0 | deleterious | 0.137 | benign | -1.383 | neutral |
|  | rs1414898839 | G45D | 0 | deleterious | 0.998 | probably damaging | -3.039 | deleterious |
|  | rs146909879 | N50S | 0.02 | deleterious | 0.918 | probably damaging | -1.763 | neutral |
|  | rs146909879 | N50T | 0.01 | deleterious | 0.963 | probably damaging | -2.541 | deleterious |
|  | rs1275353721 | G51V | 0.02 | deleterious | 0.973 | probably damaging | -4.423 | deleterious |
|  | rs181024505 | I54V | 0.29 | tolerated | 0.005 | benign | -0.317 | neutral |
|  | rs1339959525 | I54M | 0 | deleterious | 0.523 | possibly damaging | -2.097 | neutral |
|  | rs777761816 | D58A | 0 | deleterious | 0.831 | possibly damaging | -5.378 | deleterious |
|  | rs1472340973 | V59A | 0 | deleterious | 0.13 | benign | -2.839 | deleterious |
|  | rs758380334 | V59I | 0.82 | tolerated | 0.003 | benign | 0.418 | neutral |
|  | rs752738158 | I64T | 0 | deleterious | 0.272 | benign | -2.556 | deleterious |
|  | rs1446761481 | I64V | 1 | tolerated | 0.005 | benign | 0.366 | neutral |
|  | rs778728633 | V66M | 0.11 | tolerated | 0.167 | benign | -0.933 | neutral |
|  | rs1445418514 | R67T | 0 | deleterious | 0.258 | benign | -1.830 | neutral |
|  | rs753319395 | D70E | 0.7 | tolerated | 0.003 | benign | 0.867 | neutral |
|  | rs1406480386 | A71G | 0.29 | tolerated | 0.341 | benign | -1.117 | neutral |
|  | rs766004209 | F72L | 0.01 | deleterious | 0.992 | probably damaging | -4.333 | deleterious |
|  | rs1180481464 | Y73N | 0 | deleterious | 0.643 | possibly damaging | -4.167 | deleterious |
|  | rs755809863 | Y73C | 0.02 | deleterious | 0.911 | probably damaging | -4.000 | deleterious |
|  | rs147663272 | S74I | 0.17 | tolerated | 0.012 | benign | -2.697 | deleterious |
|  | rs751887116 | S74R | 0.15 | tolerated | 0.602 | possibly damaging | -1.807 | neutral |
|  | rs1274054220 | G76A | 0.03 | deleterious | 0.988 | probably damaging | -1.850 | neutral |
|  | rs763440145 | V77M | 0 | deleterious | 0.995 | probably damaging | -1.658 | neutral |
|  | rs894719968 | I84K | 0 | deleterious | 0.999 | probably damaging | -5.856 | deleterious |
|  | rs773498260 | I87T | 0.34 | tolerated | 0.006 | benign | -1.364 | neutral |
|  | rs747924098 | C88Y | 0 | deleterious | 0.993 | probably damaging | -6.044 | deleterious |
|  | rs774062845 | L92S | 0.75 | tolerated | 0.003 | benign | 1.041 | neutral |
|  | rs1035043331 | N93D | 0.88 | tolerated | 0.001 | benign | -1.020 | neutral |
|  | rs747645473 | N93T | 0.56 | tolerated | 0.171 | benign | -1.217 | neutral |
|  | rs1457120831 | T94S | 0.79 | tolerated | 0.009 | benign | 0.117 | neutral |
|  | rs1160624511 | T94A | 0.71 | tolerated | 0 | benign | 0.143 | neutral |
|  | rs1389781569 | E95K | 0.69 | tolerated | 0.001 | benign | 0.247 | neutral |
|  | rs1322983348 | V97A | 0.77 | tolerated | 0 | benign | 0.451 | neutral |
|  | rs1230385317 | V97L | 0.34 | tolerated | 0 | benign | 0.085 | neutral |
|  | rs749250079 | A99T | 0.24 | tolerated | 0 | benign | 0.034 | neutral |
|  | rs1278186978 | A100T | 0.23 | tolerated | 0.061 | benign | -0.675 | neutral |
|  | rs953791270 | A100V | 0.11 | tolerated | 0.037 | benign | -0.689 | neutral |
|  | rs1220928074 | P101S | 0.15 | tolerated | 0.001 | benign | -0.126 | neutral |
|  | rs755228476 | A104T | 0.14 | tolerated | 0.003 | benign | -0.776 | neutral |
|  | rs754257979 | L107R | 0.09 | tolerated | 0.007 | benign | -1.691 | neutral |
|  | rs780655309 | S108T | 0.29 | tolerated | 0.138 | benign | -0.798 | neutral |
|  | rs756730560 | L109V | 0.52 | tolerated | 0.028 | benign | -0.187 | neutral |
|  | rs1372298130 | T110A | 0.51 | tolerated | 0.197 | benign | -0.195 | neutral |
|  | rs748825170 | T110S | 0.16 | tolerated | 0.5 | possibly damaging | -0.500 | neutral |
|  | rs748825170 | T110N | 0.05 | tolerated | 0.773 | possibly damaging | -1.300 | neutral |
|  | rs750499540 | S111L | 0.04 | deleterious | 0.516 | possibly damaging | -1.728 | neutral |
|  | rs1166657205 | K114E | 0.17 | tolerated | 0.025 | benign | -1.355 | neutral |
|  | rs377217467 | T119S | 0.23 | tolerated | 0.027 | benign | -0.279 | neutral |
|  | rs145372366 | I120V | 1 | tolerated | 0.018 | benign | 0.056 | neutral |
|  | rs775287540 | I120N | 0.26 | tolerated | 0.708 | possibly damaging | -1.408 | neutral |
|  | rs145781517 | E121K | 0.25 | tolerated | 0.025 | benign | -0.416 | neutral |
|  | rs1339311675 | I126T | 0 | deleterious | 0.322 | benign | -2.464 | neutral |
|  | rs1378993349 | I126L | 1 | tolerated | 0 | benign | 0.116 | neutral |
|  | rs1378993349 | I126V | 0.07 | tolerated | 0.09 | benign | -0.411 | neutral |
|  | rs374308806 | I126M | 0.06 | tolerated | 0.361 | benign | -0.510 | neutral |
|  | rs1266394746 | E127D | 0.17 | tolerated | 0.735 | possibly damaging | -1.369 | neutral |
|  | rs746545019 | G129R | 0 | deleterious | 1 | probably damaging | -7.411 | deleterious |
|  | rs772697510 | T131M | 0.09 | tolerated | 0 | benign | 2.029 | neutral |
|  | rs373606965 | R133Q | 0.1 | tolerated | 0.05 | benign | -2.225 | neutral |
|  | rs1057519583 | R135T | 0 | deleterious | 0.998 | probably damaging | -4.967 | deleterious |
|  | rs1360935761 | G136D | 0 | deleterious | 1 | probably damaging | -6.389 | deleterious |
|  | rs756683816 | S137N | 0.27 | tolerated | 0.09 | benign | -0.157 | neutral |
|  | rs183917764 | R139H | 0.12 | tolerated | 0.168 | benign | -2.222 | neutral |
|  | rs781767500 | R139C | 0 | deleterious | 0.968 | probably damaging | -3.203 | deleterious |
|  | rs1056579166 | E144K | 0.07 | tolerated | 0.17 | benign | -0.646 | neutral |
|  | rs111655573 | R145Q | 0 | deleterious | 0.993 | probably damaging | -2.014 | neutral |
|  | rs559375412 | E146Q | 0 | deleterious | 0.984 | probably damaging | -1.000 | neutral |
|  | rs1355921166 | H148R | 0.45 | tolerated | 0.003 | benign | -0.013 | neutral |
|  | rs1218758426 | A149S | 0.04 | deleterious | 0.453 | possibly damaging | -2.210 | neutral |
|  | rs758696465 | A149V | 0.05 | deleterious | 0.206 | benign | -1.236 | neutral |
|  | rs1435045406 | T150I | 0.02 | deleterious | 0.243 | benign | -2.828 | deleterious |
|  | rs1435045406 | T150S | 1 | tolerated | 0.003 | benign | 0.338 | neutral |
|  | rs2487999 | T151S | 0.22 | tolerated | 0.02 | benign | -0.474 | neutral |
|  | rs2487999 | T151A | 0.64 | tolerated | 0 | benign | -0.393 | neutral |
|  | rs1467388477 | T151S | 0.22 | tolerated | 0.02 | benign | -0.474 | neutral |
|  | rs146264905 | K154E | 0 | deleterious | 0.67 | possibly damaging | -2.047 | neutral |
|  | rs1378890150 | K154N | 0 | deleterious | 0.864 | possibly damaging | -2.711 | deleterious |
|  | rs747038584 | V155A | 0 | deleterious | 0.984 | probably damaging | -2.139 | neutral |
|  | rs777948693 | D156Y | 0 | deleterious | 0.915 | probably damaging | -4.089 | deleterious |
|  | rs765462548 | D157Y | 0 | deleterious | 0.999 | probably damaging | -6.617 | deleterious |
|  | rs765462548 | D157N | 0 | deleterious | 0.996 | probably damaging | -3.444 | deleterious |
|  | rs765634171 | P158S | 0.01 | deleterious | 0.993 | probably damaging | -6.344 | deleterious |
|  | rs1210351560 | V159L | 0.07 | tolerated | 0.12 | benign | -0.834 | neutral |
|  | rs1210351560 | V159M | 0.23 | tolerated | 0.201 | benign | -0.948 | neutral |
|  | rs984613137 | I162V | 1 | tolerated | 0.003 | benign | 0.264 | neutral |
|  | rs373452798 | I164T | 0.02 | deleterious | 0.99 | probably damaging | -3.111 | deleterious |
|  | rs201407823 | R166G | 0.02 | deleterious | 0.99 | probably damaging | -3.989 | deleterious |
|  | rs1374852402 | L168F | 0.12 | tolerated | 0.955 | probably damaging | -1.903 | neutral |
|  | rs753653477 | P171S | 0 | deleterious | 0.995 | probably damaging | -4.194 | deleterious |
|  | rs1231904752 | T172S | 0.77 | tolerated | 0 | benign | 0.053 | neutral |
|  | rs1231904752 | T172A | 0.81 | tolerated | 0 | benign | -0.230 | neutral |
|  | rs1231904752 | T172P | 0.3 | tolerated | 0.072 | benign | -1.015 | neutral |
|  | rs369991097 | I173N | 0 | deleterious | 0.694 | possibly damaging | -3.684 | deleterious |
|  | rs1370240326 | I173V | 0.02 | deleterious | 0.062 | benign | -0.722 | neutral |
|  | rs188870466 | Y174C | 0 | deleterious | 0.998 | probably damaging | -5.117 | deleterious |
|  | rs1317144354 | R175G | 0 | deleterious | 0.673 | possibly damaging | -4.064 | deleterious |
|  | rs113833417 | K176R | 0.36 | tolerated | 0.011 | benign | -0.801 | neutral |
|  | rs761420179 | D179N | 0.02 | deleterious | 0.993 | probably damaging | -3.044 | deleterious |
|  | rs1228731857 | N195S | 0.39 | tolerated | 0.026 | benign | -0.906 | neutral |
|  | rs1054501193 | N195K | 0.17 | tolerated | 0.419 | benign | -1.665 | neutral |
|  | rs1054501193 | N195K | 0.17 | tolerated | 0.419 | benign | -1.665 | neutral |
|  | rs773703803 | P196L | 0.1 | tolerated | 0.265 | benign | -2.425 | neutral |
|  | rs760964853 | P196S | 0.24 | tolerated | 0.054 | benign | -0.292 | neutral |
|  | rs139991163 | A198S | 0.59 | tolerated | 0.053 | benign | 0.076 | neutral |
|  | rs139991163 | A198T | 0.72 | tolerated | 0 | benign | 0.197 | neutral |
|  | rs768856878 | A198V | 0.62 | tolerated | 0 | benign | 0.118 | neutral |
|  | rs1432455435 | D200A | 0.27 | tolerated | 0.81 | possibly damaging | -0.644 | neutral |
|  | rs981581004 | L204R | 0.09 | tolerated | 0.984 | probably damaging | -1.678 | neutral |
|  | rs1406358931 | T205S | 0.14 | tolerated | 0.457 | possibly damaging | -1.000 | neutral |
|  | rs1416450981 | T205M | 0.08 | tolerated | 0.837 | possibly damaging | -0.700 | neutral |
|  | rs1291259544 | S206N | 0.64 | tolerated | 0.001 | benign | -0.614 | neutral |
|  | rs528812123 | L207W | 0 | deleterious | 0.982 | probably damaging | -2.483 | neutral |
|  | rs200840790 | L207F | 0.07 | tolerated | 0.834 | possibly damaging | -1.522 | neutral |
|  | rs780152012 | L207V | 0.13 | tolerated | 0.03 | benign | -0.933 | neutral |
|  | rs757314485 | S209N | 0.06 | tolerated | 0.693 | possibly damaging | -0.967 | neutral |
|  | rs1483081396 | E210K | 0.06 | tolerated | 0.975 | probably damaging | -1.856 | neutral |
|  | rs377029816 | K213I | 0 | deleterious | 0.863 | possibly damaging | -3.074 | deleterious |
|  | rs1453078076 | L216H | 0 | deleterious | 1 | probably damaging | -4.222 | deleterious |
|  | rs1274653580 | M217V | 0.81 | tolerated | 0 | benign | -0.528 | neutral |
|  | rs762502692 | M217T | 0.5 | tolerated | 0.015 | benign | -1.105 | neutral |
|  | rs764804533 | E218G | 0 | deleterious | 0.74 | possibly damaging | -3.156 | deleterious |
|  | rs1289660806 | N219D | 0.01 | deleterious | 0.056 | benign | -1.700 | neutral |
|  | rs1203217688 | N219K | 0.69 | tolerated | 0.011 | benign | -0.937 | neutral |
|  | rs145933230 | N219S | 0.09 | tolerated | 0.197 | benign | -1.664 | neutral |
|  | rs773496442 | S223N | 0.19 | tolerated | 0 | benign | 0.096 | neutral |
|  | rs951926281 | F224L | 0 | deleterious | 0.999 | probably damaging | -4.100 | deleterious |
|  | rs767979237 | F224S | 0 | deleterious | 1 | probably damaging | -5.378 | deleterious |
|  | rs762370931 | Y225H | 0.11 | tolerated | 0.997 | probably damaging | -1.344 | neutral |
|  | rs1027269243 | Y225C | 0.01 | deleterious | 0.998 | probably damaging | -3.667 | deleterious |
|  | rs1389736599 | L229V | 0.11 | tolerated | 0.284 | benign | -1.650 | neutral |
|  | rs1389736599 | L229M | 0 | deleterious | 0.991 | probably damaging | -1.261 | neutral |
|  | rs768664563 | M231L | 0.47 | tolerated | 0.009 | benign | -0.206 | neutral |
|  | rs749332166 | M231T | 1 | tolerated | 0 | benign | 0.988 | neutral |
|  | rs745655678 | E233D | 0.46 | tolerated | 0.003 | benign | -0.504 | neutral |
|  | rs898925474 | E233K | 0.15 | tolerated | 0.314 | benign | -1.756 | neutral |
|  | rs1212897383 | S234F | 0.03 | deleterious | 0.947 | probably damaging | -2.900 | deleterious |
|  | rs1037819903 | L236R | 0.05 | tolerated | 0.361 | benign | -1.774 | neutral |
|  | rs781174874 | L238V | 0.07 | tolerated | 0.553 | possibly damaging | -0.811 | neutral |
|  | rs747002144 | N240S | 1 | tolerated | 0 | benign | -0.104 | neutral |
|  | rs372504367 | N240D | 0.24 | tolerated | 0 | benign | -1.169 | neutral |
|  | rs777870232 | Q241R | 0.11 | tolerated | 0.066 | benign | -1.333 | neutral |
|  | rs1431592741 | S246G | 0.21 | tolerated | 0 | benign | -1.099 | neutral |
|  | rs1310985753 | S246R | 0.12 | tolerated | 0.001 | benign | -1.354 | neutral |
|  | rs10786775 | S248C | 0.61 | tolerated | 0 | benign | 0.860 | neutral |
|  | rs10786775 | S248Y | 0.04 | deleterious | 0.146 | benign | -1.148 | neutral |
|  | rs767922406 | D250N | 0.36 | tolerated | 0 | benign | -1.223 | neutral |
|  | rs1335241545 | V252M | 0.13 | tolerated | 0.055 | benign | -0.810 | neutral |
|  | rs766023768 | N253T | 0.41 | tolerated | 0.061 | benign | -0.792 | neutral |
|  | rs573295762 | N253D | 0.98 | tolerated | 0 | benign | -0.024 | neutral |
|  | rs1253432306 | F254I | 0.39 | tolerated | 0.003 | benign | 0.100 | neutral |
|  | rs553430081 | K255E | 0.22 | tolerated | 0 | benign | -0.810 | neutral |
|  | rs1045409658 | T259P | 0.04 | deleterious | 0.306 | benign | -1.713 | neutral |
|  | rs757577860 | K261E | 0.02 | deleterious | 0.138 | benign | -1.385 | neutral |
|  | rs751941982 | A262T | 0.49 | tolerated | 0.138 | benign | -0.361 | neutral |
|  | rs764639049 | A262E | 0.85 | tolerated | 0.258 | benign | 0.482 | neutral |
|  | rs1312355950 | H264Y | 0.15 | tolerated | 0.874 | possibly damaging | -1.811 | neutral |
|  | rs367699574 | H264R | 0.42 | tolerated | 0.062 | benign | -1.275 | neutral |
|  | rs139258012 | S265N | 0.16 | tolerated | 0 | benign | -0.696 | neutral |
|  | rs74157365 | S265R | 0.12 | tolerated | 0.001 | benign | -1.302 | neutral |
|  | rs1461332009 | I266T | 0.03 | deleterious | 0.572 | possibly damaging | -2.393 | neutral |
|  | rs146867381 | A270V | 0.08 | tolerated | 0.966 | probably damaging | -1.595 | neutral |
|  | rs201612020 | I271V | 0.08 | tolerated | 0.046 | benign | -0.429 | neutral |
|  | rs201612020 | I271L | 0.5 | tolerated | 0.028 | benign | -0.164 | neutral |
|  | rs201076579 | Q275R | 0.09 | tolerated | 0.024 | benign | -1.674 | neutral |
|  | rs965987401 | E276Q | 0.02 | deleterious | 0.379 | benign | -1.129 | neutral |
|  | rs1250352727 | G278R | 0 | deleterious | 1 | probably damaging | -5.856 | deleterious |
|  | rs772038898 | L279R | 0.05 | deleterious | 0.669 | possibly damaging | -2.135 | neutral |
|  | rs199709485 | V280A | 0 | deleterious | 0.772 | possibly damaging | -2.528 | deleterious |
|  | rs142616199 | Q282R | 0.39 | tolerated | 0.995 | probably damaging | -1.528 | neutral |
|  | rs748826736 | K283E | 0 | deleterious | 0.492 | possibly damaging | -1.973 | neutral |
|  | rs779633657 | K283N | 0 | deleterious | 0.76 | possibly damaging | -2.677 | deleterious |
|  | rs1337433407 | D284G | 0.39 | tolerated | 0.088 | benign | -1.349 | neutral |
|  | rs1304733420 | G286C | 0.09 | tolerated | 0.639 | possibly damaging | -2.411 | neutral |
|  | rs1449271366 | D288H | 0.02 | deleterious | 0.04 | benign | -1.292 | neutral |
|  | rs1390771835 | Y291C | 0.04 | deleterious | 0.953 | probably damaging | -5.089 | deleterious |
|  | rs1019246020 | Y291H | 0 | deleterious | 0.419 | benign | -3.256 | deleterious |
|  | rs1036189827 | Y292C | 0.11 | tolerated | 0.096 | benign | -2.549 | deleterious |
|  | rs1009255770 | T294I | 0 | deleterious | 0.986 | probably damaging | -2.792 | deleterious |
|  | rs1230760638 | E296K | 0.32 | tolerated | 0.015 | benign | 0.072 | neutral |
|  | rs1297860081 | D299E | 0.96 | tolerated | 0 | benign | -0.879 | neutral |
|  | rs1453655282 | L300V | 0 | deleterious | 0.989 | probably damaging | -1.929 | neutral |
|  | rs1396259698 | L300P | 0 | deleterious | 0.999 | probably damaging | -4.324 | deleterious |
|  | rs1195859795 | H301R | 0.37 | tolerated | 0.335 | benign | -2.531 | deleterious |
|  | rs779578647 | H301Q | 0.31 | tolerated | 0.018 | benign | -1.998 | neutral |
|  | rs1252786688 | I304N | 0 | deleterious | 0.715 | possibly damaging | -3.206 | deleterious |
|  | rs753557631 | R306W | 0.07 | tolerated | 0.663 | possibly damaging | -1.439 | neutral |
|  | rs745579048 | R306Q | 0.45 | tolerated | 0.001 | benign | 0.676 | neutral |
|  | rs541666260 | I308L | 0.08 | tolerated | 0.279 | benign | -0.897 | neutral |
|  | rs1224932955 | Q309E | 0.02 | deleterious | 0.334 | benign | -0.996 | neutral |
|  | rs1053718163 | C312R | 0 | deleterious | 0.826 | possibly damaging | -5.351 | deleterious |
|  | rs753139908 | C312F | 0 | deleterious | 0.924 | probably damaging | -4.805 | deleterious |
|  | rs1339657049 | P315A | 0.02 | deleterious | 0.999 | probably damaging | -3.123 | deleterious |
|  | rs374196630 | N316S | 0.05 | tolerated | 0.185 | benign | -1.002 | neutral |
|  | rs1206780644 | H317N | 0 | deleterious | 0.313 | benign | -2.969 | deleterious |
|  | rs1269188009 | M318I | 0.09 | tolerated | 0 | benign | -0.160 | neutral |
|  | rs756219814 | C322Y | 0 | deleterious | 0.999 | probably damaging | -6.494 | deleterious |
|  | rs1449612751 | H323Q | 0 | deleterious | 0.999 | probably damaging | -4.323 | deleterious |
|  | rs202001347 | F324L | 0.33 | tolerated | 0.007 | benign | -1.465 | neutral |
|  | rs202001347 | F324I | 0.14 | tolerated | 0.234 | benign | -1.521 | neutral |
|  | rs767610473 | F324L | 0.33 | tolerated | 0.007 | benign | -1.465 | neutral |
|  | rs757540440 | H326P | 0 | deleterious | 0.999 | probably damaging | -5.356 | deleterious |
|  | rs763936289 | I327V | 0.3 | tolerated | 0.068 | benign | -0.257 | neutral |
|  | rs763936289 | I327L | 0 | deleterious | 0.357 | benign | -1.106 | neutral |
|  | rs762750326 | I327T | 0.02 | deleterious | 0.824 | possibly damaging | -2.310 | neutral |
|  | rs758924832 | R332C | 0 | deleterious | 0.828 | possibly damaging | -3.901 | deleterious |
|  | rs758924832 | R332S | 0 | deleterious | 0.392 | benign | -2.462 | neutral |
|  | rs770464178 | R332L | 0.02 | deleterious | 0.013 | benign | -3.088 | deleterious |
|  | rs770464178 | R332H | 0.12 | tolerated | 0.007 | benign | -1.275 | neutral |
|  | rs746747175 | L333V | 0.19 | tolerated | 0.793 | possibly damaging | -0.796 | neutral |
|  | rs1474827122 | L333R | 0.06 | tolerated | 0.767 | possibly damaging | -0.492 | neutral |
|  | rs761593090 | R336C | 0.15 | tolerated | 0.17 | benign | -0.670 | neutral |
|  | rs115811576 | R336H | 0.31 | tolerated | 0 | benign | 0.017 | neutral |
|  | rs780655414 | P337L | 0.52 | tolerated | 0.47 | possibly damaging | -1.787 | neutral |
|  | rs757406867 | E341K | 0.41 | tolerated | 0.012 | benign | -0.409 | neutral |
|  | rs1379046613 | A342S | 0.06 | tolerated | 0.086 | benign | -0.453 | neutral |
|  | rs1379046613 | A342P | 0.06 | tolerated | 0.003 | benign | -1.240 | neutral |
|  | rs751748363 | Q345H | 0.47 | tolerated | 0.548 | possibly damaging | -1.474 | neutral |
|  | rs776552307 | E349K | 0.29 | tolerated | 0.115 | benign | -1.032 | neutral |
|  | rs934067959 | L350H | 0.12 | tolerated | 0.98 | probably damaging | -1.799 | neutral |
|  | rs1280873028 | D353A | 0.58 | tolerated | 0.162 | benign | -1.356 | neutral |
|  | rs1353836308 | Q354H | 0.02 | deleterious | 0.365 | benign | -1.213 | neutral |
|  | rs370342283 | S355G | 0 | deleterious | 0.985 | probably damaging | -2.695 | deleterious |
|  | rs200376630 | I357T | 0 | deleterious | 0.36 | benign | -2.677 | deleterious |
|  | rs1460949175 | M361I | 0.08 | tolerated | 0.015 | benign | -1.351 | neutral |
|  | rs758150440 | M361V | 0.19 | tolerated | 0.015 | benign | -1.186 | neutral |
|  | rs1053016616 | H363R | 0.61 | tolerated | 0 | benign | -0.480 | neutral |
|  | rs765139782 | H363Y | 0.04 | deleterious | 0.013 | benign | -1.457 | neutral |
|  | rs1196643886 | Y364C | 0.02 | deleterious | 0.998 | probably damaging | -3.874 | deleterious |
|  | rs1468540767 | Y365N | 0 | deleterious | 0.998 | probably damaging | -4.946 | deleterious |
|  | rs935996262 | Y365C | 0 | deleterious | 0.998 | probably damaging | -5.113 | deleterious |
|  | rs753286739 | T366A | 0.12 | tolerated | 0 | benign | -1.387 | neutral |
|  | rs140449924 | A367V | 0.17 | tolerated | 0.058 | benign | -1.156 | neutral |

**Table S2:** Structure-based predictions of destabilizing mutations in *STN1* gene.

| **S. No.** | **Variants** | **mCSM** | | **SDM2** | | **DUET** | | **CUPSAT** | | **STRUM** | |
| --- | --- | --- | --- | --- | --- | --- | --- | --- | --- | --- | --- |
|  |  | **Score** | **Remarks** | **Score** | **Remarks** | **Score** | **Remarks** | **Score** | **Remarks** | **Score** | **Remarks** |
|  | L24P | -0.737 | Destabilizing | 0.0 | Destabilizing | -0.438 | Destabilizing | 3.58 | Stabilizing | -1.7 | Destabilizing |
|  | A25G | -0.382 | Destabilizing | 0.14 | Stabilizing | -0.031 | Destabilizing | 0.35 | Stabilizing | -0.97 | Destabilizing |
|  | F26L | -1.156 | Destabilizing | -0.11 | Destabilizing | -1.003 | Destabilizing | -0.45 | Destabilizing | -1.61 | Destabilizing |
|  | K28E | -0.54 | Destabilizing | -0.06 | Destabilizing | -0.195 | Destabilizing | -0.26 | Destabilizing | -0.58 | Destabilizing |
|  | L29H | -2.734 | Destabilizing | -0.7 | Destabilizing | -2.623 | Destabilizing | 1.88 | Stabilizing | -2.13 | Destabilizing |
|  | L35R | -0.72 | Destabilizing | -0.7 | Destabilizing | -0.5 | Destabilizing | -1.64 | Destabilizing | -0.7 | Destabilizing |
|  | D36N | -0.346 | Destabilizing | 0.09 | Stabilizing | -0.024 | Destabilizing | -0.51 | Destabilizing | -0.16 | Destabilizing |
|  | M37V | -1.428 | Destabilizing | -0.62 | Destabilizing | -1.261 | Destabilizing | -2.58 | Destabilizing | -0.85 | Destabilizing |
|  | K38T | -0.187 | Destabilizing | 0.1 | Stabilizing | 0.206 | Stabilizing | 1.15 | Stabilizing | -0.03 | Destabilizing |
|  | R41S | -0.005 | Destabilizing | -0.65 | Destabilizing | 0.038 | Stabilizing | -0.29 | Destabilizing | -1.66 | Destabilizing |
|  | Q42H | 0.075 | Stabilizing | 0.7 | Stabilizing | 0.352 | Stabilizing | -1.34 | Destabilizing | -0.37 | Destabilizing |
|  | V43L | -0.297 | Destabilizing | -0.24 | Destabilizing | -0.155 | Destabilizing | -0.92 | Destabilizing | -1.82 | Destabilizing |
|  | G45D | -0.763 | Destabilizing | -2.8 | Destabilizing | -1.124 | Destabilizing | -3.85 | Destabilizing | -0.84 | Destabilizing |
|  | N50S | -0.979 | Destabilizing | -1.18 | Destabilizing | -1.105 | Destabilizing | -1.07 | Destabilizing | -0.74 | Destabilizing |
|  | N50T | -0.686 | Destabilizing | -0.63 | Destabilizing | -0.632 | Destabilizing | -0.37 | Destabilizing | -1.11 | Destabilizing |
|  | G51V | -0.325 | Destabilizing | -2.25 | Destabilizing | -0.65 | Destabilizing | -2.38 | Destabilizing | -1.4 | Destabilizing |
|  | I54V | -1.511 | Destabilizing | -2.38 | Destabilizing | -1.817 | Destabilizing | -1.66 | Destabilizing | -0.43 | Destabilizing |
|  | I54M | -0.775 | Destabilizing | -1.38 | Destabilizing | -0.913 | Destabilizing | -3.57 | Destabilizing | -0.49 | Destabilizing |
|  | D58A | -1.022 | Destabilizing | 1.65 | Stabilizing | -0.34 | Destabilizing | -5.96 | Destabilizing | -0.55 | Destabilizing |
|  | V59A | -2.365 | Destabilizing | -2.66 | Destabilizing | -2.184 | Destabilizing | -3.94 | Destabilizing | -1.42 | Destabilizing |
|  | V59I | -0.868 | Destabilizing | -0.6 | Destabilizing | -0.642 | Destabilizing | -3.22 | Destabilizing | -0.93 | Destabilizing |
|  | I64T | -1.212 | Destabilizing | -1.05 | Destabilizing | -0.997 | Destabilizing | 2.82 | Stabilizing | -1.25 | Destabilizing |
|  | I64V | -0.902 | Destabilizing | 0.19 | Stabilizing | -0.473 | Destabilizing | 2.53 | Stabilizing | -0.87 | Destabilizing |
|  | V66M | -0.399 | Destabilizing | -1.38 | Destabilizing | -0.499 | Destabilizing | -1.65 | Destabilizing | -0.79 | Destabilizing |
|  | R67T | -0.113 | Destabilizing | -0.05 | Destabilizing | 0.153 | Stabilizing | 3.53 | Stabilizing | -1.34 | Destabilizing |
|  | D70E | -0.206 | Destabilizing | 0.01 | Stabilizing | 0.139 | Stabilizing | 1.22 | Stabilizing | -0.96 | Destabilizing |
|  | A71G | -0.91 | Destabilizing | -0.51 | Destabilizing | -0.767 | Destabilizing | -0.04 | Destabilizing | -1.49 | Destabilizing |
|  | F72L | -0.869 | Destabilizing | 0.11 | Stabilizing | -0.734 | Destabilizing | -2.91 | Destabilizing | -1.28 | Destabilizing |
|  | Y73N | -2.32 | Destabilizing | -2.21 | Destabilizing | -2.515 | Destabilizing | 0.07 | Stabilizing | -1.24 | Destabilizing |
|  | Y73C | -1.229 | Destabilizing | -1.16 | Destabilizing | -1.276 | Destabilizing | -0.73 | Destabilizing | -0.9 | Destabilizing |
|  | S74I | 0.028 | Stabilizing | 1.17 | Stabilizing | 0.63 | Stabilizing | 2.55 | Stabilizing | 0.3 | Stabilizing |
|  | S74R | -0.436 | Destabilizing | -0.33 | Destabilizing | -0.1 | Destabilizing | 1.74 | Stabilizing | 0.1 | Stabilizing |
|  | G76A | -0.802 | Destabilizing | -1.17 | Destabilizing | -0.718 | Destabilizing | -0.5 | Destabilizing | -0.14 | Destabilizing |
|  | V77M | -1.419 | Destabilizing | -2.05 | Destabilizing | -1.802 | Destabilizing | -5.04 | Destabilizing | -1.51 | Destabilizing |
|  | I84K | -1.827 | Destabilizing | -3.56 | Destabilizing | -2.1 | Destabilizing | -6.51 | Destabilizing | -1.99 | Destabilizing |
|  | I87T | -2.516 | Destabilizing | -1.74 | Destabilizing | -2.546 | Destabilizing | -4.39 | Destabilizing | -1.04 | Destabilizing |
|  | C88Y | -0.384 | Destabilizing | -1.09 | Destabilizing | -0.423 | Destabilizing | -3.36 | Destabilizing | -2.19 | Destabilizing |
|  | L92S | -0.453 | Destabilizing | -1.5 | Destabilizing | -0.386 | Destabilizing | -0.48 | Destabilizing | -1.48 | Destabilizing |
|  | N93D | -0.264 | Destabilizing | 0.06 | Stabilizing | 0.105 | Stabilizing | -0.64 | Destabilizing | -0.48 | Destabilizing |
|  | N93T | -0.49 | Destabilizing | -0.09 | Destabilizing | -0.166 | Destabilizing | -0.62 | Destabilizing | -0.9 | Destabilizing |
|  | T94S | -1.226 | Destabilizing | -1.1 | Destabilizing | -1.238 | Destabilizing | -2.1 | Destabilizing | -0.68 | Destabilizing |
|  | T94A | -1.221 | Destabilizing | 1.49 | Stabilizing | -0.632 | Destabilizing | -1.74 | Destabilizing | -0.78 | Destabilizing |
|  | E95K | 0.196 | Stabilizing | -0.46 | Destabilizing | 0.443 | Stabilizing | -0.41 | Destabilizing | -1.55 | Destabilizing |
|  | V97A | -0.536 | Destabilizing | -0.36 | Destabilizing | -0.309 | Destabilizing | -0.67 | Destabilizing | -1.75 | Destabilizing |
|  | V97L | -0.264 | Destabilizing | 0.04 | Stabilizing | 0.07 | Stabilizing | -0.69 | Destabilizing | -1.54 | Destabilizing |
|  | A99T | -0.679 | Destabilizing | -0.31 | Destabilizing | -0.383 | Destabilizing | 2.56 | Stabilizing | -0.08 | Destabilizing |
|  | A100T | -0.794 | Destabilizing | -2.03 | Destabilizing | -0.845 | Destabilizing | -0.11 | Destabilizing | -1.05 | Destabilizing |
|  | A100V | -0.442 | Destabilizing | -0.99 | Destabilizing | -0.358 | Destabilizing | -2.09 | Destabilizing | -1.3 | Destabilizing |
|  | P101S | -0.538 | Destabilizing | -0.84 | Destabilizing | -0.34 | Destabilizing | -0.4 | Destabilizing | -0.92 | Destabilizing |
|  | A104T | -1.004 | Destabilizing | -1.33 | Destabilizing | -0.863 | Destabilizing | 1.26 | Destabilizing | -0.22 | Destabilizing |
|  | L107R | -0.275 | Destabilizing | -0.8 | Destabilizing | -0.126 | Destabilizing | -2.23 | Destabilizing | -1.06 | Destabilizing |
|  | S108T | -0.316 | Destabilizing | 0.76 | Stabilizing | 0.171 | Stabilizing | -0.51 | Destabilizing | -0.45 | Destabilizing |
|  | L109V | -1.093 | Destabilizing | 0.12 | Stabilizing | -0.789 | Destabilizing | -0.21 | Destabilizing | -1.05 | Destabilizing |
|  | T110A | -0.419 | Destabilizing | 0.32 | Stabilizing | -0.249 | Destabilizing | -0.58 | Destabilizing | -0.71 | Destabilizing |
|  | T110S | -0.415 | Destabilizing | -0.73 | Destabilizing | -0.4 | Destabilizing | -0.34 | Destabilizing | -0.54 | Destabilizing |
|  | T110N | -0.151 | Destabilizing | 0.16 | Stabilizing | 0.059 | Stabilizing | 0.86 | Stabilizing | -0.15 | Destabilizing |
|  | S111L | -0.042 | Destabilizing | 1.24 | Stabilizing | 0.425 | Stabilizing | -0.95 | Destabilizing | -0.38 | Destabilizing |
|  | K114E | -0.122 | Destabilizing | 0.35 | Stabilizing | 0.041 | Stabilizing | 0.56 | Stabilizing | -0.19 | Destabilizing |
|  | T119S | -0.746 | Destabilizing | -0.46 | Destabilizing | -0.485 | Destabilizing | -0.31 | Destabilizing | -0.72 | Destabilizing |
|  | I120V | -0.584 | Destabilizing | -0.18 | Destabilizing | -0.238 | Destabilizing | 0.26 | Stabilizing | -1.35 | Destabilizing |
|  | I120N | -0.239 | Destabilizing | -0.48 | Destabilizing | 0.031 | Stabilizing | 0.06 | Stabilizing | -1.48 | Destabilizing |
|  | E121K | -0.04 | Destabilizing | -0.89 | Destabilizing | 0.185 | Stabilizing | 0.22 | Stabilizing | -1.44 | Destabilizing |
|  | I126T | -2.402 | Destabilizing | -1.94 | Destabilizing | -2.519 | Destabilizing | 1.45 | Stabilizing | -1.84 | Destabilizing |
|  | I126L | -1.033 | Destabilizing | -0.02 | Destabilizing | -0.759 | Destabilizing | -1.25 | Destabilizing | -1.44 | Destabilizing |
|  | I126V | -1.479 | Destabilizing | -2.61 | Destabilizing | -1.864 | Destabilizing | -0.04 | Destabilizing | -1.43 | Destabilizing |
|  | I126M | -1.002 | Destabilizing | -0.75 | Destabilizing | -1.065 | Destabilizing | -0.23 | Destabilizing | -1.49 | Destabilizing |
|  | E127D | -0.491 | Destabilizing | -0.4 | Destabilizing | -0.401 | Destabilizing | -0.59 | Destabilizing | -0.88 | Destabilizing |
|  | G129R | -0.521 | Destabilizing | -2.32 | Destabilizing | -0.737 | Destabilizing | -1.53 | Destabilizing | -1.24 | Destabilizing |
|  | T131M | -0.078 | Destabilizing | -0.78 | Destabilizing | -0.164 | Destabilizing | -0.3 | Destabilizing | -2.07 | Destabilizing |
|  | R133Q | -0.994 | Destabilizing | -0.73 | Destabilizing | -0.919 | Destabilizing | -2.87 | Destabilizing | 0.35 | Stabilizing |
|  | R135T | -1.773 | Destabilizing | -0.25 | Destabilizing | -1.555 | Destabilizing | 4.9 | Stabilizing | -1.0 | Destabilizing |
|  | G136D | -1.987 | Destabilizing | -2.08 | Destabilizing | -2.097 | Destabilizing | -2.36 | Destabilizing | -0.01 | Destabilizing |
|  | S137N | -0.924 | Destabilizing | 0.02 | Stabilizing | -0.553 | Destabilizing | 2.07 | Stabilizing | -0.42 | Destabilizing |
|  | R139H | -1.617 | Destabilizing | 0.52 | Stabilizing | -1.268 | Destabilizing | -2.09 | Destabilizing | -0.98 | Destabilizing |
|  | R139C | -0.933 | Destabilizing | 0.1 | Stabilizing | -0.733 | Destabilizing | 2.23 | Stabilizing | -1.49 | Destabilizing |
|  | E144K | 0.113 | Stabilizing | -0.43 | Destabilizing | 0.484 | Stabilizing | 0.51 | Stabilizing | -1.44 | Destabilizing |
|  | R145Q | -0.879 | Destabilizing | -1.14 | Destabilizing | -0.928 | Destabilizing | -3.07 | Destabilizing | -1.47 | Destabilizing |
|  | E146Q | -1.883 | Destabilizing | -1.08 | Destabilizing | -1.875 | Destabilizing | -3.52 | Destabilizing | 0.61 | Stabilizing |
|  | H148R | -1.184 | Destabilizing | -0.3 | Destabilizing | -1.028 | Destabilizing | -1.98 | Destabilizing | -0.56 | Destabilizing |
|  | A149S | -1.489 | Destabilizing | -2.32 | Destabilizing | -1.565 | Destabilizing | 1.05 | Stabilizing | -1.28 | Destabilizing |
|  | A149V | -0.3 | Destabilizing | 0.95 | Stabilizing | 0.345 | Stabilizing | 0.97 | Stabilizing | -1.18 | Destabilizing |
|  | T150I | -0.26 | Destabilizing | 1.21 | Stabilizing | 0.352 | Stabilizing | -1.54 | Destabilizing | -0.27 | Destabilizing |
|  | T150S | -0.433 | Destabilizing | -1.37 | Destabilizing | -0.363 | Destabilizing | 0.35 | Stabilizing | -0.9 | Destabilizing |
|  | T151S | -0.694 | Destabilizing | -1.37 | Destabilizing | -0.651 | Destabilizing | -0.1 | Destabilizing | -0.52 | Destabilizing |
|  | T151A | -0.803 | Destabilizing | -0.31 | Destabilizing | -0.646 | Destabilizing | -1.43 | Destabilizing | -0.52 | Destabilizing |
|  | T151S | -0.694 | Destabilizing | -1.37 | Destabilizing | -0.651 | Destabilizing | 0.35 | Destabilizing | -0.52 | Destabilizing |
|  | K154E | 0.246 | Stabilizing | -0.1 | Destabilizing | 0.598 | Stabilizing | 0.01 | Stabilizing | -0.1 | Destabilizing |
|  | K154N | -0.382 | Destabilizing | -0.55 | Destabilizing | -0.168 | Destabilizing | -1.51 | Destabilizing | -0.35 | Destabilizing |
|  | V155A | -1.761 | Destabilizing | -2.07 | Destabilizing | -2.022 | Destabilizing | -0.97 | Destabilizing | -0.79 | Destabilizing |
|  | D156Y | 0.163 | Stabilizing | 0.26 | Stabilizing | 0.251 | Stabilizing | -3.71 | Destabilizing | -2.03 | Destabilizing |
|  | D157Y | -0.031 | Destabilizing | -1.33 | Destabilizing | -0.518 | Destabilizing | -1.98 | Destabilizing | -0.91 | Destabilizing |
|  | D157N | -1.308 | Destabilizing | -0.37 | Destabilizing | -1.291 | Destabilizing | -1.6 | Destabilizing | 0.13 | Stabilizing |
|  | P158S | -0.512 | Destabilizing | -0.26 | Destabilizing | -0.339 | Destabilizing | -0.21 | Destabilizing | -1.09 | Destabilizing |
|  | V159L | -0.303 | Destabilizing | 0.11 | Stabilizing | -0.056 | Destabilizing | 0.17 | Stabilizing | -2.05 | Destabilizing |
|  | V159M | -0.522 | Destabilizing | -0.35 | Destabilizing | -0.561 | Destabilizing | -1.87 | Destabilizing | -1.84 | Destabilizing |
|  | I162V | -1.158 | Destabilizing | -0.35 | Destabilizing | -0.906 | Destabilizing | 0.76 | Stabilizing | -0.64 | Destabilizing |
|  | I164T | -1.123 | Destabilizing | -1.03 | Destabilizing | -0.949 | Destabilizing | -0.52 | Destabilizing | -0.91 | Destabilizing |
|  | R166G | -1.782 | Destabilizing | -3.32 | Destabilizing | -2.522 | Destabilizing | -0.26 | Destabilizing | -0.91 | Destabilizing |
|  | L168F | -0.75 | Destabilizing | -0.57 | Destabilizing | -0.786 | Destabilizing | -0.06 | Destabilizing | -1.07 | Destabilizing |
|  | P171S | -1.749 | Destabilizing | -0.23 | Destabilizing | -1.436 | Destabilizing | -4.05 | Destabilizing | -0.97 | Destabilizing |
|  | T172S | -0.822 | Destabilizing | -0.26 | Destabilizing | -0.516 | Destabilizing | 0.58 | Stabilizing | -0.8 | Destabilizing |
|  | T172A | -0.595 | Destabilizing | 1.53 | Stabilizing | 0.118 | Stabilizing | 1.57 | Stabilizing | -0.45 | Destabilizing |
|  | T172P | -0.182 | Destabilizing | -0.27 | Destabilizing | 0.063 | Stabilizing | -1.91 | Destabilizing | -0.78 | Destabilizing |
|  | I173N | -2.092 | Destabilizing | -0.96 | Destabilizing | -1.956 | Destabilizing | -3.1 | Destabilizing | -0.79 | Destabilizing |
|  | I173V | -1.161 | Destabilizing | -0.35 | Destabilizing | -0.909 | Destabilizing | -1.61 | Destabilizing | -0.94 | Destabilizing |
|  | Y174C | -0.872 | Destabilizing | -1.43 | Destabilizing | -0.867 | Destabilizing | -2.11 | Destabilizing | -1.16 | Destabilizing |
|  | R175G | -0.572 | Destabilizing | -0.85 | Destabilizing | -0.618 | Destabilizing | -0.77 | Destabilizing | -1.02 | Destabilizing |
|  | K176R | -0.099 | Destabilizing | -0.11 | Destabilizing | 0.226 | Stabilizing | 0.05 | Stabilizing | -0.01 | Destabilizing |
|  | D179N | -1.183 | Destabilizing | 0.16 | Stabilizing | -1.075 | Destabilizing | -1.16 | Destabilizing | -0.58 | Destabilizing |
|  | N195S | -0.068 | Destabilizing | -1.05 | Destabilizing | 0.023 | Stabilizing | 1.04 | Stabilizing | -0.81 | Destabilizing |
|  | N195K | 0.165 | Stabilizing | -0.46 | Destabilizing | 0.441 | Stabilizing | 3.84 | Stabilizing | -1.01 | Destabilizing |
|  | N195K | 0.165 | Stabilizing | -0.46 | Destabilizing | 0.441 | Stabilizing | 3.84 | Stabilizing | -1.01 | Destabilizing |
|  | P196L | -0.609 | Destabilizing | 2.12 | Stabilizing | 0.216 | Stabilizing | 2.32 | Stabilizing | -0.76 | Destabilizing |
|  | P196S | -1.802 | Destabilizing | -0.59 | Destabilizing | -1.562 | Destabilizing | 0.12 | Stabilizing | -0.86 | Destabilizing |
|  | A198S | -0.42 | Destabilizing | -1.99 | Destabilizing | -0.377 | Destabilizing | -1.33 | Destabilizing | -0.73 | Destabilizing |
|  | A198T | -0.499 | Destabilizing | -1.53 | Destabilizing | -0.369 | Destabilizing | -0.94 | Destabilizing | -0.32 | Destabilizing |
|  | A198V | -0.213 | Destabilizing | -1.03 | Destabilizing | -0.047 | Destabilizing | -0.48 | Destabilizing | -0.55 | Destabilizing |
|  | D200A | -0.302 | Destabilizing | 0.11 | Stabilizing | -0.132 | Destabilizing | -0.41 | Destabilizing | -0.79 | Destabilizing |
|  | L204R | -2.148 | Destabilizing | -3.02 | Destabilizing | -2.216 | Destabilizing | -5.23 | Destabilizing | -1.1 | Destabilizing |
|  | T205S | -1.306 | Destabilizing | -0.99 | Destabilizing | -1.239 | Destabilizing | -1.51 | Destabilizing | -0.45 | Destabilizing |
|  | T205M | -0.368 | Destabilizing | 0.32 | Stabilizing | -0.122 | Destabilizing | 062 | Stabilizing | -0.77 | Destabilizing |
|  | S206N | -0.514 | Destabilizing | 0.65 | Stabilizing | -0.016 | Destabilizing | -0.28 | Destabilizing | -0.5 | Destabilizing |
|  | L207W | -1.615 | Destabilizing | -0.56 | Destabilizing | -1.474 | Destabilizing | 0.24 | Stabilizing | -3.01 | Destabilizing |
|  | L207F | -1.472 | Destabilizing | -0.69 | Destabilizing | -1.508 | Destabilizing | -0.21 | Destabilizing | -1.75 | Destabilizing |
|  | L207V | -1.332 | Destabilizing | -0.61 | Destabilizing | -1.167 | Destabilizing | -1.88 | Destabilizing | -1.76 | Destabilizing |
|  | S209N | -1.215 | Destabilizing | -0.23 | Destabilizing | -0.958 | Destabilizing | -1.17 | Destabilizing | -0.01 | Destabilizing |
|  | E210K | -0.252 | Destabilizing | -0.77 | Destabilizing | -0.012 | Destabilizing | 0.86 | Stabilizing | -0.91 | Destabilizing |
|  | K213I | 0.419 | Stabilizing | 0.62 | Stabilizing | 0.841 | Stabilizing | -0.42 | Destabilizing | -0.73 | Destabilizing |
|  | L216H | -2.717 | Destabilizing | -1.94 | Destabilizing | -2.884 | Destabilizing | 0.55 | Stabilizing | -1.39 | Destabilizing |
|  | M217V | -1.666 | Destabilizing | 0.2 | Stabilizing | -1.155 | Destabilizing | -0.57 | Destabilizing | -1.19 | Destabilizing |
|  | M217T | -1.407 | Destabilizing | -0.98 | Destabilizing | -1.085 | Destabilizing | -1.86 | Destabilizing | -1.56 | Destabilizing |
|  | E218G | -0.836 | Destabilizing | -1.22 | Destabilizing | -0.909 | Destabilizing | -0.53 | Destabilizing | -0.66 | Destabilizing |
|  | N219D | 0.367 | Stabilizing | -0.83 | Destabilizing | 0.407 | Stabilizing | 0.28 | Stabilizing | -0.23 | Destabilizing |
|  | N219K | 0.144 | Stabilizing | -0.15 | Destabilizing | 0.414 | Stabilizing | 0.89 | Stabilizing | -0.61 | Destabilizing |
|  | N219S | -0.28 | Destabilizing | -1.26 | Destabilizing | -0.342 | Destabilizing | -0.78 | Destabilizing | -0.74 | Destabilizing |
|  | S223N | -0.039 | Destabilizing | 0.57 | Stabilizing | 0.493 | Stabilizing | -2.19 | Destabilizing | -0.34 | Destabilizing |
|  | F224L | -0.382 | Destabilizing | -2.39 | Destabilizing | -0.659 | Destabilizing | -5.31 | Destabilizing | -1.8 | Destabilizing |
|  | F224S | -2.966 | Destabilizing | -3.53 | Destabilizing | -3.259 | Destabilizing | -3.04 | Destabilizing | -2.56 | Destabilizing |
|  | Y225H | -0.836 | Destabilizing | -0.09 | Destabilizing | -0.472 | Destabilizing | 1.16 | Stabilizing | -1.25 | Destabilizing |
|  | Y225C | -0.775 | Destabilizing | -0.11 | Destabilizing | -0.609 | Destabilizing | 7.35 | Stabilizing | -1.11 | Destabilizing |
|  | L229V | -1.145 | Destabilizing | -3.21 | Destabilizing | -1.599 | Destabilizing | -1.97 | Destabilizing | -0.82 | Destabilizing |
|  | L229M | -0.622 | Destabilizing | -0.99 | Destabilizing | -0.649 | Destabilizing | -1.59 | Destabilizing | -0.6 | Destabilizing |
|  | M231L | -0.103 | Destabilizing | 0.3 | Stabilizing | 0.354 | Stabilizing | 0.6 | Stabilizing | -0.63 | Destabilizing |
|  | M231T | 0.039 | Stabilizing | -0.69 | Destabilizing | 0.337 | Stabilizing | 3.01 | Stabilizing | -1.32 | Destabilizing |
|  | E233D | -0.273 | Destabilizing | -1.42 | Destabilizing | -0.218 | Destabilizing | -0.31 | Destabilizing | -0.06 | Destabilizing |
|  | E233K | 0.399 | Stabilizing | -1.0 | Destabilizing | 0.612 | Stabilizing | -0.19 | Destabilizing | -0.79 | Destabilizing |
|  | S234F | -1.039 | Destabilizing | 0.76 | Stabilizing | -0.662 | Destabilizing | 0.01 | Stabilizing | -0.45 | Destabilizing |
|  | L236R | -1.162 | Destabilizing | -0.7 | Destabilizing | -0.931 | Destabilizing | 1.03 | Stabilizing | -1.25 | Destabilizing |
|  | L238V | -1.414 | Destabilizing | -3.1 | Destabilizing | -1.866 | Destabilizing | -1.31 | Destabilizing | -0.64 | Destabilizing |
|  | N240S | -0.75 | Destabilizing | -0.97 | Destabilizing | -0.773 | Destabilizing | -0.73 | Destabilizing | -0.82 | Destabilizing |
|  | N240D | -0.334 | Destabilizing | -0.14 | Destabilizing | -0.116 | Destabilizing | 0.3 | Stabilizing | 0.08 | Stabilizing |
|  | Q241R | -0.47 | Destabilizing | -0.44 | Destabilizing | -0.328 | Destabilizing | -0.65 | Destabilizing | -0.66 | Destabilizing |
|  | S246G | -0.237 | Destabilizing | 0.85 | Stabilizing | 0.308 | Stabilizing | 0.42 | Stabilizing | -1.1 | Destabilizing |
|  | S246R | -0.133 | Destabilizing | 1.4 | Stabilizing | 0.421 | Stabilizing | 0.54 | Stabilizing | -0.12 | Destabilizing |
|  | S248C | -0.092 | Destabilizing | 1.02 | Stabilizing | 0.346 | Stabilizing | -1.47 | Destabilizing | -0.8 | Destabilizing |
|  | S248Y | -0.921 | Destabilizing | 0.73 | Stabilizing | -0.566 | Destabilizing | -1.75 | Destabilizing | -0.4 | Destabilizing |
|  | D250N | 0.45 | Stabilizing | 0.07 | Stabilizing | 0.657 | Stabilizing | 0.76 | Stabilizing | -0.57 | Destabilizing |
|  | V252M | -0.38 | Destabilizing | -0.35 | Destabilizing | -0.407 | Destabilizing | 2.52 | Stabilizing | -1.14 | Destabilizing |
|  | N253T | -0.039 | Destabilizing | -0.16 | Destabilizing | 0.184 | Stabilizing | -1.24 | Destabilizing | -0.43 | Destabilizing |
|  | N253D | 0.17 | Stabilizing | 0.07 | Stabilizing | 0.455 | Stabilizing | -0.32 | Destabilizing | 0.06 | Stabilizing |
|  | F254I | -0.779 | Destabilizing | 0.56 | Stabilizing | -0.55 | Destabilizing | 1.04 | Stabilizing | -1.51 | Destabilizing |
|  | K255E | -0.024 | Destabilizing | 1.0 | Stabilizing | 0.566 | Stabilizing | 0.17 | Stabilizing | -0.1 | Destabilizing |
|  | T259P | -0.415 | Destabilizing | -0.35 | Destabilizing | -0.207 | Destabilizing | 0.6 | Stabilizing | -1.42 | Destabilizing |
|  | K261E | -0.837 | Destabilizing | 0.23 | Stabilizing | -0.422 | Destabilizing | -0.73 | Destabilizing | -0.61 | Destabilizing |
|  | A262T | -1.58 | Destabilizing | -3.32 | Destabilizing | -1.878 | Destabilizing | -1.37 | Destabilizing | -0.38 | Destabilizing |
|  | A262E | -2.193 | Destabilizing | -2.94 | Destabilizing | -2.501 | Destabilizing | -0.28 | Destabilizing | -0.41 | Destabilizing |
|  | H264Y | 0.439 | Stabilizing | 0.14 | Stabilizing | 0.694 | Stabilizing | 0.65 | Stabilizing | -0.88 | Destabilizing |
|  | H264R | -1.061 | Destabilizing | -0.19 | Destabilizing | -0.851 | Destabilizing | -0.14 | Destabilizing | 0.36 | Stabilizing |
|  | S265N | -0.936 | Destabilizing | 0.62 | Stabilizing | -0.461 | Destabilizing | -0.56 | Destabilizing | -0.29 | Destabilizing |
|  | S265R | -0.752 | Destabilizing | 1.44 | Stabilizing | -0.161 | Destabilizing | 1.06 | Stabilizing | 0.07 | Stabilizing |
|  | I266T | -2.723 | Destabilizing | -3.46 | Destabilizing | -3.053 | Destabilizing | -2.61 | Destabilizing | -1.49 | Destabilizing |
|  | A270V | -0.649 | Destabilizing | -1.03 | Destabilizing | -0.526 | Destabilizing | 1.55 | Stabilizing | -0.44 | Destabilizing |
|  | I271V | -1.684 | Destabilizing | -2.85 | Destabilizing | -2.107 | Destabilizing | -0.56 | Destabilizing | -1.48 | Destabilizing |
|  | I271L | -1.073 | Destabilizing | 0.23 | Stabilizing | -0.66 | Destabilizing | 0.57 | Stabilizing | -1.42 | Destabilizing |
|  | Q275R | -0.774 | Destabilizing | 0.42 | Stabilizing | -0.361 | Destabilizing | -1.89 | Destabilizing | 0.74 | Stabilizing |
|  | E276Q | -0.229 | Destabilizing | -1.14 | Destabilizing | -0.11 | Destabilizing | -0.71 | Destabilizing | -0.55 | Destabilizing |
|  | G278R | -1.033 | Destabilizing | -3.43 | Destabilizing | -1.374 | Destabilizing | -4.55 | Destabilizing | -0.69 | Destabilizing |
|  | L279R | -1.388 | Destabilizing | -1.45 | Destabilizing | -1.309 | Destabilizing | -1.33 | Destabilizing | -1.14 | Destabilizing |
|  | V280A | -2.14 | Destabilizing | -2.66 | Destabilizing | -2.572 | Destabilizing | -2.68 | Destabilizing | -0.75 | Destabilizing |
|  | Q282R | -0.066 | Destabilizing | 0.55 | Stabilizing | 0.284 | Stabilizing | -0.88 | Destabilizing | -0.13 | Destabilizing |
|  | K283E | -0.493 | Destabilizing | -0.31 | Destabilizing | -0.294 | Destabilizing | -0.33 | Destabilizing | -0.82 | Destabilizing |
|  | K283N | -0.953 | Destabilizing | -0.31 | Destabilizing | -0.811 | Destabilizing | -0.85 | Destabilizing | -0.78 | Destabilizing |
|  | D284G | 0.041 | Stabilizing | 0.59 | Stabilizing | 0.419 | Stabilizing | 0.07 | Stabilizing | -1.21 | Destabilizing |
|  | G286C | -0.928 | Destabilizing | -1.62 | Destabilizing | -1.068 | Destabilizing | 2.18 | Stabilizing | -1.2 | Destabilizing |
|  | D288H | 0.205 | Stabilizing | 0.04 | Stabilizing | 0.172 | Stabilizing | -2.33 | Destabilizing | -1.17 | Destabilizing |
|  | Y291C | -1.577 | Destabilizing | -1.04 | Destabilizing | -1.62 | Destabilizing | -3.33 | Destabilizing | -1.22 | Destabilizing |
|  | Y291H | -2.127 | Destabilizing | -1.15 | Destabilizing | -2.115 | Destabilizing | -5.14 | Destabilizing | -1.12 | Destabilizing |
|  | Y292C | -1.214 | Destabilizing | 0.06 | Stabilizing | -1.029 | Destabilizing | -5.0 | Destabilizing | -0.85 | Destabilizing |
|  | T294I | -0.006 | Destabilizing | 0.49 | Stabilizing | 0.327 | Stabilizing | 0.73 | Stabilizing | -0.45 | Destabilizing |
|  | E296K | -0.921 | Destabilizing | -1.1 | Destabilizing | -1.009 | Destabilizing | -1.02 | Destabilizing | -1.33 | Destabilizing |
|  | D299E | -0.584 | Destabilizing | 1.24 | Stabilizing | 0.097 | Stabilizing | 0.65 | Stabilizing | -0.8 | Destabilizing |
|  | L300V | -1.754 | Destabilizing | -3.21 | Destabilizing | -2.254 | Destabilizing | -1.27 | Destabilizing | -1.46 | Destabilizing |
|  | L300P | -1.754 | Destabilizing | -4.68 | Destabilizing | -2.48 | Destabilizing | -5.25 | Destabilizing | -2.25 | Destabilizing |
|  | H301R | -1.578 | Destabilizing | -1.84 | Destabilizing | -1.65 | Destabilizing | 0.24 | Stabilizing | -1.13 | Destabilizing |
|  | H301Q | -1.235 | Destabilizing | -1.27 | Destabilizing | -1.268 | Destabilizing | 1.23 | Stabilizing | -1.47 | Destabilizing |
|  | I304N | -3.403 | Destabilizing | -2.63 | Destabilizing | -3.511 | Destabilizing | 1.94 | Stabilizing | -1.35 | Destabilizing |
|  | R306W | -0.205 | Destabilizing | -0.32 | Destabilizing | -0.441 | Destabilizing | -1.52 | Destabilizing | -1.44 | Destabilizing |
|  | R306Q | -0.112 | Destabilizing | -0.17 | Destabilizing | 0.132 | Stabilizing | -0.55 | Destabilizing | -1.07 | Destabilizing |
|  | I308L | -1.294 | Destabilizing | 0.23 | Stabilizing | -0.899 | Destabilizing | -1.31 | Destabilizing | -0.95 | Destabilizing |
|  | Q309E | -0.801 | Destabilizing | 0.77 | Stabilizing | -0.198 | Destabilizing | 0.68 | Stabilizing | -0.29 | Destabilizing |
|  | C312R | -1.307 | Destabilizing | -0.86 | Destabilizing | -1.022 | Destabilizing | -1.89 | Destabilizing | -0.63 | Destabilizing |
|  | C312F | -1.056 | Destabilizing | -0.39 | Destabilizing | -0.907 | Destabilizing | -1.27 | Destabilizing | -1.44 | Destabilizing |
|  | P315A | -0.597 | Destabilizing | -0.2 | Destabilizing | -0.466 | Destabilizing | 1.19 | Stabilizing | -0.96 | Destabilizing |
|  | N316S | -0.056 | Destabilizing | -0.77 | Destabilizing | 0.01 | Stabilizing | -2.35 | Destabilizing | -0.37 | Destabilizing |
|  | H317N | 0.651 | Stabilizing | -0.16 | Destabilizing | 0.559 | Stabilizing | -1.94 | Destabilizing | -1.93 | Destabilizing |
|  | M318I | -0.728 | Destabilizing | 0.63 | Stabilizing | -0.267 | Destabilizing | 3.69 | Stabilizing | -0.5 | Destabilizing |
|  | C322Y | -1.375 | Destabilizing | -1.54 | Destabilizing | -1.498 | Destabilizing | -4.92 | Destabilizing | -1.12 | Destabilizing |
|  | H323Q | -1.333 | Destabilizing | -0.71 | Destabilizing | -1.316 | Destabilizing | 2.77 | Stabilizing | -1.38 | Destabilizing |
|  | F324L | -1.678 | Destabilizing | -1.72 | Destabilizing | -1.829 | Destabilizing | -1.58 | Destabilizing | -1.07 | Destabilizing |
|  | F324I | -1.678 | Destabilizing | -0.13 | Destabilizing | -1.536 | Destabilizing | -1.17 | Destabilizing | -0.74 | Destabilizing |
|  | F324L | -1.678 | Destabilizing | -1.72 | Destabilizing | -1.829 | Destabilizing | -1.58 | Destabilizing | -1.07 | Destabilizing |
|  | H326P | 0.216 | Stabilizing | -1.92 | Destabilizing | -0.064 | Destabilizing | -5.1 | Destabilizing | -0.63 | Destabilizing |
|  | I327V | -1.755 | Destabilizing | -2.85 | Destabilizing | -2.184 | Destabilizing | -1.19 | Destabilizing | -1.39 | Destabilizing |
|  | I327L | -1.179 | Destabilizing | 0.23 | Stabilizing | -0.774 | Destabilizing | 0.63 | Stabilizing | -1.23 | Destabilizing |
|  | I327T | -3.205 | Destabilizing | -2.8 | Destabilizing | -3.439 | Destabilizing | -1.3 | Destabilizing | -1.87 | Destabilizing |
|  | R332C | -1.153 | Destabilizing | -0.5 | Destabilizing | -1.092 | Destabilizing | -0.98 | Destabilizing | -0.86 | Destabilizing |
|  | R332S | -1.437 | Destabilizing | -2.05 | Destabilizing | -1.735 | Destabilizing | -1.61 | Destabilizing | -1.17 | Destabilizing |
|  | R332L | -0.387 | Destabilizing | 0.49 | Stabilizing | -0.018 | Destabilizing | -1.38 | Destabilizing | -0.6 | Destabilizing |
|  | R332H | -1.617 | Destabilizing | -0.09 | Destabilizing | -1.502 | Destabilizing | -2.38 | Destabilizing | -0.82 | Destabilizing |
|  | L333V | -0.25 | Destabilizing | -0.39 | Destabilizing | 0.066 | Stabilizing | -0.09 | Destabilizing | -1.06 | Destabilizing |
|  | L333R | 0.352 | Stabilizing | 0.09 | Stabilizing | 0.603 | Stabilizing | 0.24 | Stabilizing | -1.02 | Destabilizing |
|  | R336C | -1.103 | Destabilizing | 0.08 | Stabilizing | -1.064 | Destabilizing | -0.03 | Destabilizing | -1.02 | Destabilizing |
|  | R336H | -1.362 | Destabilizing | 0.58 | Stabilizing | -1.205 | Destabilizing | 0.05 | Stabilizing | -1.46 | Destabilizing |
|  | P337L | -0.234 | Destabilizing | 0.84 | Stabilizing | 0.178 | Stabilizing | 1.45 | Stabilizing | -1.28 | Destabilizing |
|  | E341K | 0.433 | Stabilizing | -0.98 | Destabilizing | 0.651 | Stabilizing | -0.16 | Destabilizing | -0.32 | Destabilizing |
|  | A342S | -1.337 | Destabilizing | -2.75 | Destabilizing | -1.526 | Destabilizing | -0.68 | Destabilizing | -0.4 | Destabilizing |
|  | A342P | -0.774 | Destabilizing | -3.0 | Destabilizing | -1.152 | Destabilizing | 2.42 | Stabilizing | 0.04 | Stabilizing |
|  | Q345H | -0.925 | Destabilizing | 0.11 | Stabilizing | -0.716 | Destabilizing | -0.91 | Destabilizing | -0.34 | Destabilizing |
|  | E349K | -0.674 | Destabilizing | -0.58 | Destabilizing | -0.422 | Destabilizing | 0.23 | Stabilizing | -0.28 | Destabilizing |
|  | L350H | -1.85 | Destabilizing | -0.79 | Destabilizing | -1.786 | Destabilizing | -2.71 | Destabilizing | -1.36 | Destabilizing |
|  | D353A | -0.373 | Destabilizing | 0.95 | Stabilizing | 0.164 | Stabilizing | -0.13 | Destabilizing | -0.78 | Destabilizing |
|  | Q354H | -0.646 | Destabilizing | 1.02 | Stabilizing | -0.353 | Destabilizing | -0.77 | Destabilizing | -0.1 | Destabilizing |
|  | S355G | -0.677 | Destabilizing | 3.74 | Stabilizing | 0.312 | Stabilizing | 0.05 | Stabilizing | -0.53 | Destabilizing |
|  | I357T | -3.056 | Destabilizing | -3.36 | Destabilizing | -3.349 | Destabilizing | -1.83 | Destabilizing | -1.18 | Destabilizing |
|  | M361I | 0.023 | Stabilizing | 0.8 | Stabilizing | 0.765 | Stabilizing | -2.29 | Destabilizing | -0.9 | Destabilizing |
|  | M361V | -0.072 | Destabilizing | 1.01 | Stabilizing | 0.711 | Stabilizing | -0.87 | Destabilizing | -0.93 | Destabilizing |
|  | H363R | -0.887 | Destabilizing | -0.28 | Destabilizing | -0.741 | Destabilizing | -1.1 | Destabilizing | -0.55 | Destabilizing |
|  | H363Y | 1.008 | Stabilizing | -0.04 | Destabilizing | 1.048 | Stabilizing | -1.09 | Destabilizing | -1.47 | Destabilizing |
|  | Y364C | -1.561 | Destabilizing | -1.22 | Destabilizing | -1.637 | Destabilizing | 1.96 | Stabilizing | -0.91 | Destabilizing |
|  | Y365N | -2.991 | Destabilizing | -1.96 | Destabilizing | -3.148 | Destabilizing | -6.41 | Destabilizing | -1.44 | Destabilizing |
|  | Y365C | -1.597 | Destabilizing | -1.02 | Destabilizing | -1.637 | Destabilizing | -3.92 | Destabilizing | -1.26 | Destabilizing |
|  | T366A | -1.27 | Destabilizing | 0.03 | Stabilizing | -1.055 | Destabilizing | -2.06 | Destabilizing | -0.17 | Destabilizing |
|  | A367V | -0.614 | Destabilizing | 1.66 | Stabilizing | 0.175 | Stabilizing | 2.13 | Stabilizing | -0.31 | Destabilizing |

**Table S3:** Association of deleterious/destabilizing mutations with the phenotype.

| **S. No.** | **Mutation** | **MutPred2** | | **PHD-SNP** | |
| --- | --- | --- | --- | --- | --- |
|  |  | **Score** | **Remarks** | **Score** | **Remarks** |
|  | L24P | 0.681 | Pathogenic | 8 | Disease |
|  | A25G | 0.402 | benign | 3 | Neutral |
|  | F26L | 0.692 | Pathogenic | 5 | Disease |
|  | K28E | 0.598 | Pathogenic | 1 | Disease |
|  | L29H | 0.814 | Pathogenic | 3 | Disease |
|  | L35R | 0.865 | Pathogenic | 7 | Disease |
|  | G45D | 0.857 | Pathogenic | 5 | Disease |
|  | N50S | 0.259 | benign | 3 | Neutral |
|  | N50T | 0.419 | benign | 4 | Disease |
|  | G51V | 0.632 | Pathogenic | 7 | Disease |
|  | I54M | 0.576 | Pathogenic | 3 | Disease |
|  | D58A | 0.617 | Pathogenic | 0 | Neutral |
|  | V59A | 0.442 | benign | 7 | Neutral |
|  | I64T | 0.699 | Pathogenic | 1 | Neutral |
|  | F72L | 0.659 | Pathogenic | 3 | Disease |
|  | Y73N | 0.665 | Pathogenic | 6 | Disease |
|  | Y73C | 0.461 | benign | 1 | Disease |
|  | G76A | 0.470 | benign | 2 | Neutral |
|  | V77M | 0.455 | benign | 2 | Neutral |
|  | I84K | 0.885 | Pathogenic | 7 | Disease |
|  | C88Y | 0.594 | Pathogenic | 6 | Disease |
|  | S111L | 0.231 | benign | 7 | Neutral |
|  | G129R | 0.846 | Pathogenic | 2 | Neutral |
|  | R135T | 0.626 | Pathogenic | 1 | Disease |
|  | G136D | 0.887 | Pathogenic | 1 | Neutral |
|  | R139C | 0.343 | benign | 2 | Neutral |
|  | R145Q | 0.390 | benign | 3 | Disease |
|  | E146Q | 0.593 | Pathogenic | 3 | Neutral |
|  | A149S | 0.389 | benign | 2 | Disease |
|  | T150I | 0.223 | benign | 1 | Neutral |
|  | K154N | 0.413 | benign | 2 | Neutral |
|  | V155A | 0.393 | benign | 0 | Neutral |
|  | D157Y | 0.683 | Pathogenic | 9 | Disease |
|  | D157N | 0.334 | benign | 5 | Disease |
|  | P158S | 0.552 | Pathogenic | 0 | Disease |
|  | I164T | 0.433 | benign | 4 | Disease |
|  | R166G | 0.741 | Pathogenic | 2 | Disease |
|  | L168F | 0.178 | benign | 8 | Neutral |
|  | P171S | 0.479 | benign | 2 | Neutral |
|  | I173N | 0.606 | Pathogenic | 3 | Disease |
|  | Y174C | 0.627 | Pathogenic | 5 | Disease |
|  | R175G | 0.316 | benign | 0 | Disease |
|  | D179N | 0.373 | benign | 2 | Neutral |
|  | L207W | 0.240 | benign | 4 | Neutral |
|  | L216H | 0.699 | Pathogenic | 5 | Disease |
|  | E218G | 0.454 | benign | 6 | Neutral |
|  | F224L | 0.497 | benign | 1 | Neutral |
|  | F224S | 0.717 | Pathogenic | 6 | Disease |
|  | Y225C | 0.413 | benign | 1 | Neutral |
|  | L229M | 0.240 | benign | 6 | Neutral |
|  | S234F | 0.240 | benign | 6 | Neutral |
|  | I266T | 0.201 | benign | 5 | Neutral |
|  | G278R | 0.852 | Pathogenic | 3 | Disease |
|  | L279R | 0.549 | Pathogenic | 4 | Neutral |
|  | V280A | 0.517 | Pathogenic | 6 | Neutral |
|  | K283E | 0.396 | benign | 5 | Neutral |
|  | K283N | 0.335 | benign | 1 | Neutral |
|  | Y291C | 0.749 | Pathogenic | 3 | Disease |
|  | Y291H | 0.628 | Pathogenic | 5 | Neutral |
|  | L300V | 0.382 | benign | 0 | Neutral |
|  | L300P | 0.844 | Pathogenic | 6 | Disease |
|  | I304N | 0.692 | Pathogenic | 2 | Disease |
|  | C312R | 0.710 | Pathogenic | 2 | Disease |
|  | C312F | 0.645 | Pathogenic | 5 | Disease |
|  | P315A | 0.184 | benign | 5 | Neutral |
|  | H317N | 0.125 | benign | 5 | Neutral |
|  | C322Y | 0.779 | Pathogenic | 8 | Disease |
|  | H323Q | 0.494 | benign | 1 | Disease |
|  | H326P | 0.774 | Pathogenic | 5 | Disease |
|  | I327T | 0.549 | Pathogenic | 4 | Neutral |
|  | R332C | 0.241 | benign | 1 | Neutral |
|  | R332L | 0.302 | benign | 0 | Disease |
|  | I357T | 0.470 | benign | 2 | Disease |
|  | Y364C | 0.256 | benign | 3 | Disease |
|  | Y365N | 0.868 | Pathogenic | 6 | Disease |
|  | Y365C | 0.824 | Pathogenic | 4 | Disease |

**Table S4:** The calculated parameters for all the system obtained after 100 ns MD simulations.

| **S. No.** | **Protein** | **Average**  **RMSD (nm)** | **RMSF (nm)** | **Radius of gyration (nm)** | **Average**  **SASA (nm^2^)** | **Average**  **No. of HB** | **Volume (nm^3^)** | **Density**  **(kg/m^3^)** | **Kinetic Energy (kJ/mol)** | **Average**  **Potential Energy (kJ/mol)** | **Enthalpy (kJ/mol)** | **Total Energy**  **(kJ/mol)** |
| --- | --- | --- | --- | --- | --- | --- | --- | --- | --- | --- | --- | --- |
| **1.** | **STN1wt** | 0.64 | 0.19 | 1.59 | 92.42 | 114 | 517.58 | 1015.31 | 127925 | -794587 | -666630 | -666661 |
| **2.** | **STN1 D157Y** | 0.62 | 0.31 | 1.83 | 99.02 | 105 | 517.79 | 1015.23 | 127967 | -794394 | -666396 | -666427 |
| **3.** | **STN1 D157Y (Run 2)** | 0.68 | 0.28 | 1.79 | 95.38 | 106 | 520.93 | 1015.02 | 128738 | -799187 | -667417 | -668448 |
| **4.** | **STN1 R135T** | 0.60 | 0.21 | 1.61 | 93.43 | 106 | 517.54 | 1015.27 | 127913 | -794457 | -666513 | -666544 |
