# Supplementary material for: Structural Analysis and Conformational Dynamics of STN1 Gene Mutations Involved in Coat Plus Syndrome
Source: Front Mol Biosci. 2019 Jun 12;6:41. doi: 10.3389/fmolb.2019.00041 (PMC6581698; doi:10.3389/fmolb.2019.00041)
Supplement: Supplementary file 2 [file Presentation_1.PPTX]

## Slide 1
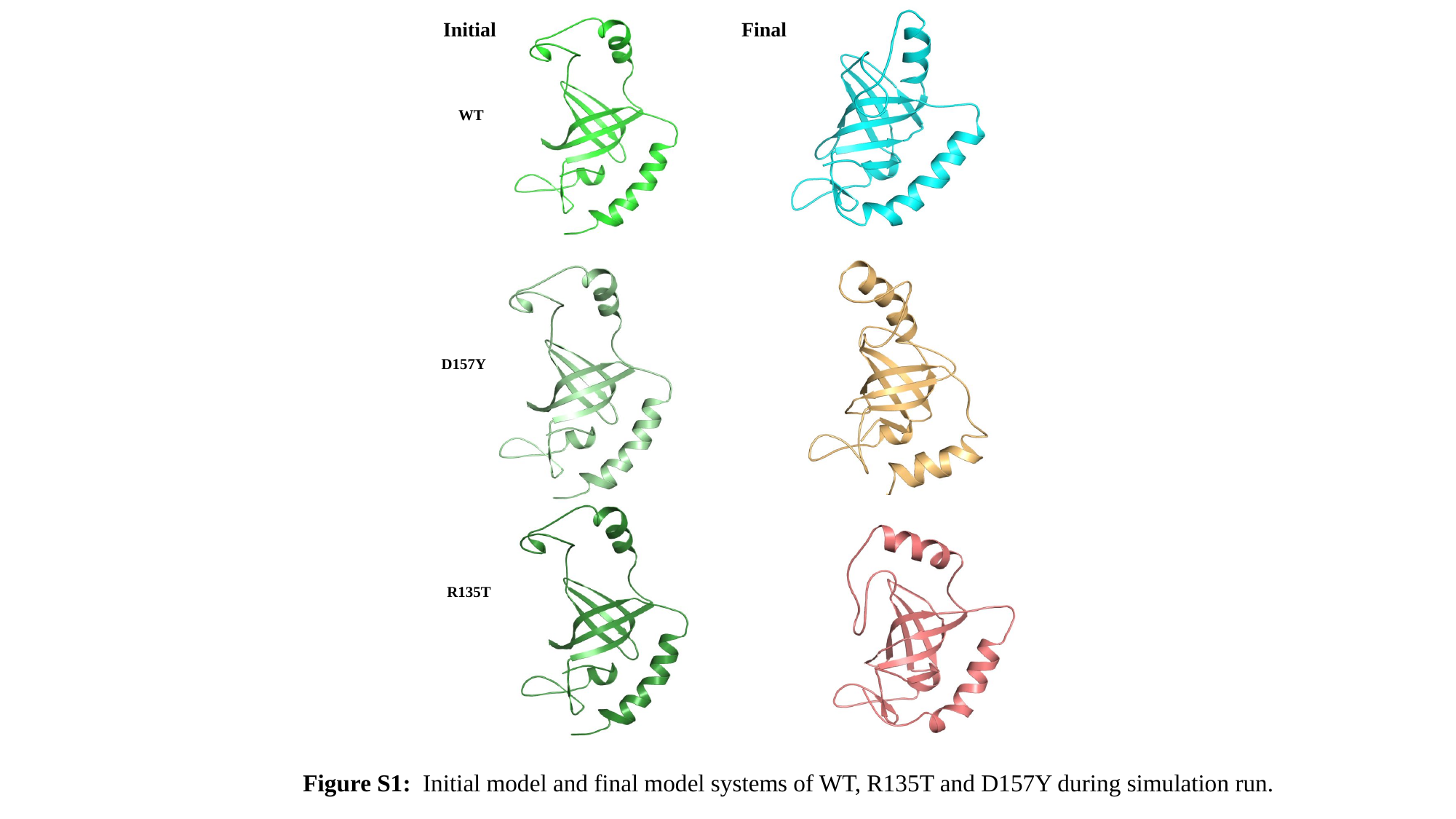

Final
Initial
WT
D157Y
R135T
Figure S1: Initial model and final model systems of WT, R135T and D157Y during simulation run.

## Slide 2
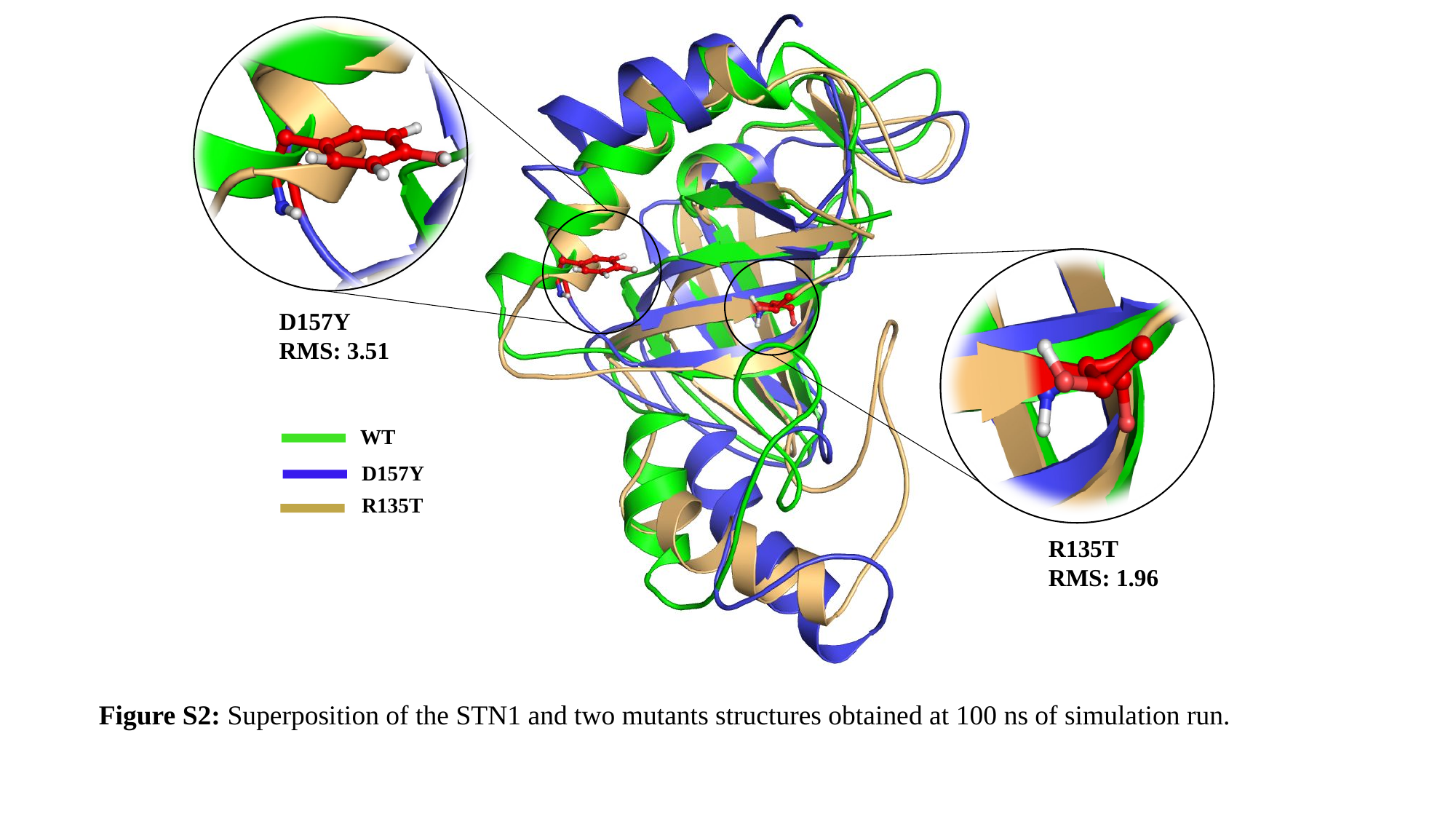

D157Y
RMS: 3.51
WT
D157Y
R135T
R135T
RMS: 1.96
Figure S2: Superposition of the STN1 and two mutants structures obtained at 100 ns of simulation run.

## Slide 3
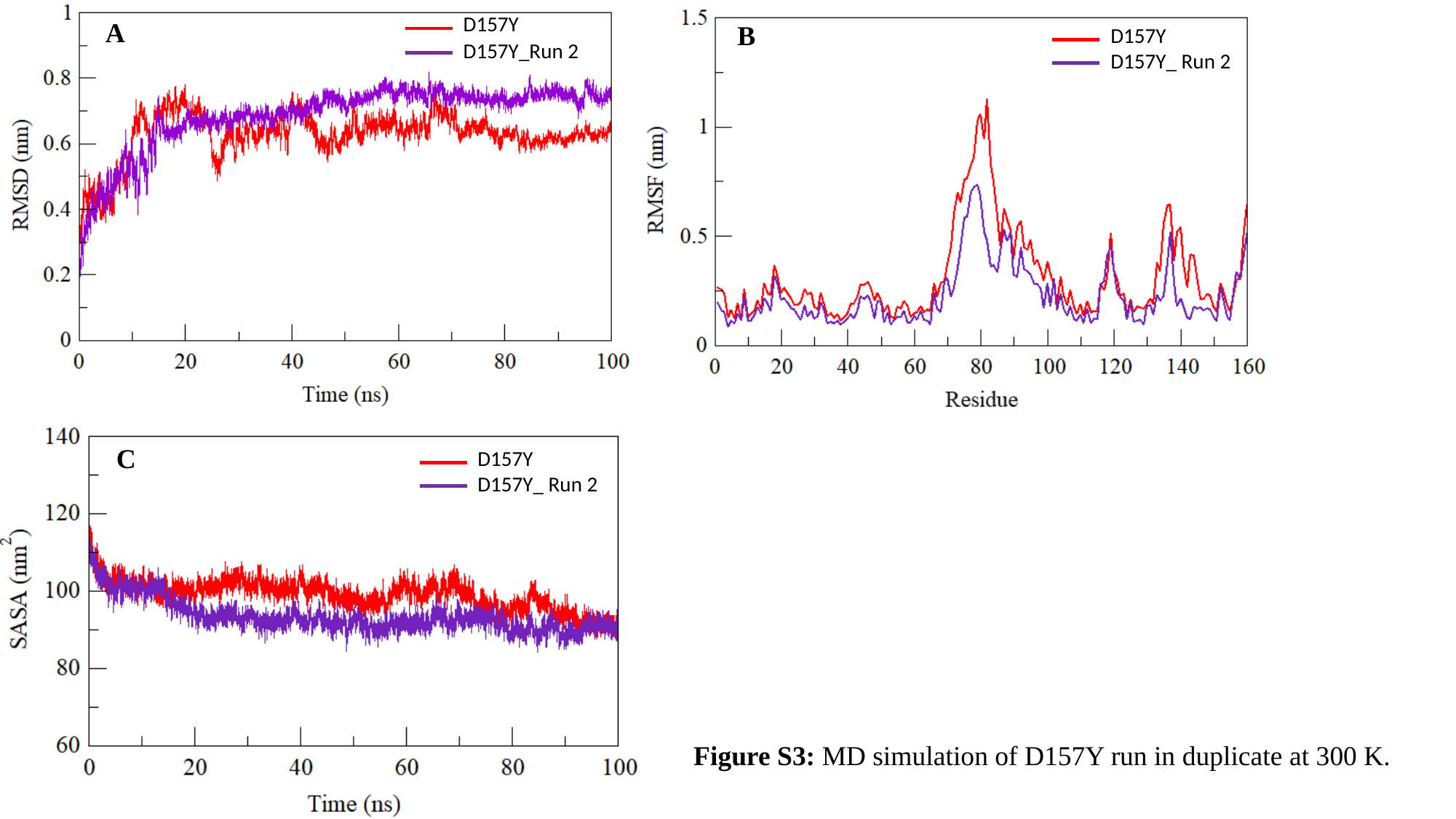

D157Y
A
B
D157Y
D157Y_Run 2
D157Y_ Run 2
C
D157Y
D157Y_ Run 2
Figure S3: MD simulation of D157Y run in duplicate at 300 K.

## Slide 4
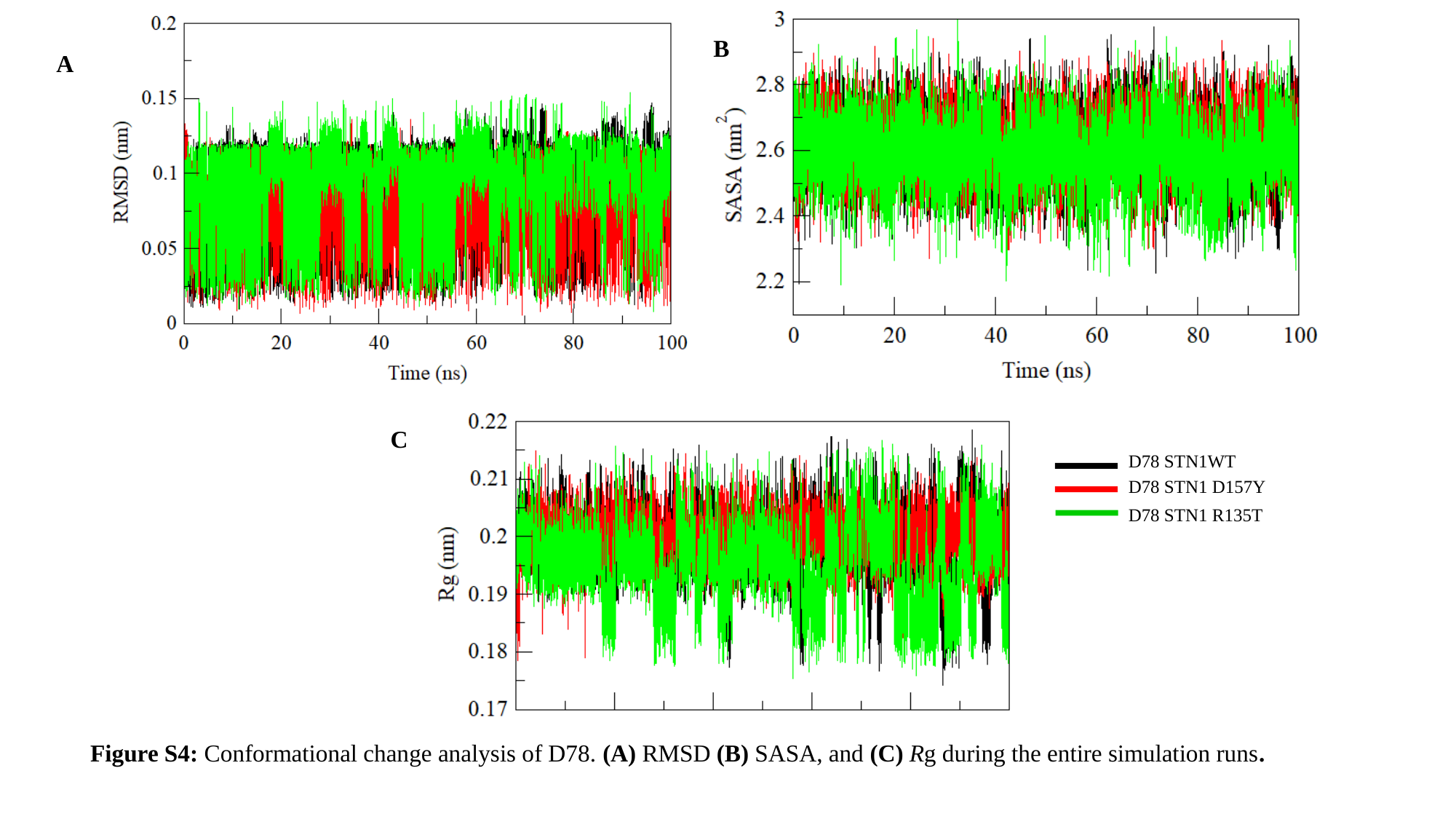

B
A
C
D78 STN1WT
D78 STN1 D157Y
D78 STN1 R135T
Figure S4: Conformational change analysis of D78. (A) RMSD (B) SASA, and (C) Rg during the entire simulation runs.
